# Gene panel testing of 5589 *BRCA1/2*‐negative index patients with breast cancer in a routine diagnostic setting: results of the German Consortium for Hereditary Breast and Ovarian Cancer

**DOI:** 10.1002/cam4.1376

**Published:** 2018-03-09

**Authors:** Jan Hauke, Judit Horvath, Eva Groß, Andrea Gehrig, Ellen Honisch, Karl Hackmann, Gunnar Schmidt, Norbert Arnold, Ulrike Faust, Christian Sutter, Julia Hentschel, Shan Wang‐Gohrke, Mateja Smogavec, Bernhard H. F. Weber, Nana Weber‐Lassalle, Konstantin Weber‐Lassalle, Julika Borde, Corinna Ernst, Janine Altmüller, Alexander E. Volk, Holger Thiele, Verena Hübbel, Peter Nürnberg, Katharina Keupp, Beatrix Versmold, Esther Pohl, Christian Kubisch, Sabine Grill, Victoria Paul, Natalie Herold, Nadine Lichey, Kerstin Rhiem, Nina Ditsch, Christian Ruckert, Barbara Wappenschmidt, Bernd Auber, Andreas Rump, Dieter Niederacher, Thomas Haaf, Juliane Ramser, Bernd Dworniczak, Christoph Engel, Alfons Meindl, Rita K. Schmutzler, Eric Hahnen

**Affiliations:** ^1^ Center for Hereditary Breast and Ovarian Cancer Center for Integrated Oncology (CIO) Medical Faculty University Hospital Cologne Cologne Germany; ^2^ Institute for Human Genetics University Hospital Muenster Muenster Germany; ^3^ Department of Gynaecology and Obstetrics Division of Tumor Genetics Klinikum rechts der Isar Technical University Munich Munich Germany; ^4^ Institute of Human Genetics Julius‐Maximilians‐Universität Würzburg Würzburg Germany; ^5^ Department of Gynaecology and Obstetrics University Hospital Duesseldorf Heinrich‐Heine University Duesseldorf Duesseldorf Germany; ^6^ Institute for Clinical Genetics Technische Universitaet Dresden Dresden Germany; ^7^ Department of Human Genetics Hannover Medical School Hannover Germany; ^8^ Department of Gynaecology and Obstetrics Institute of Clinical Molecular Biology University Hospital of Schleswig‐Holstein, Campus Kiel Christian‐Albrechts University Kiel Kiel Germany; ^9^ Institute of Medical Genetics and Applied Genomics University Hospital Tuebingen Tuebingen Germany; ^10^ Institute of Human Genetics University of Heidelberg Heidelberg Germany; ^11^ Institute of Human Genetics University of Leipzig Hospitals and Clinics Leipzig Germany; ^12^ Department of Gynaecology and Obstetrics University Hospital Ulm Ulm Germany; ^13^ Institute of Human Genetics University Medical Center Georg August University Goettingen Germany; ^14^ Institute of Human Genetics University of Regensburg Regensburg Germany; ^15^ Cologne Center for Genomics University of Cologne Cologne Germany; ^16^ Institute of Human Genetics University of Cologne Cologne Germany; ^17^ Cologne Excellence Cluster on Cellular Stress Responses in Aging‐Associated Diseases (CECAD) University of Cologne Cologne Germany; ^18^ Institute of Human Genetics University Medical Center Hamburg‐Eppendorf Hamburg Germany; ^19^ Department of Obstetrics and Gynaecology Ludwig‐Maximilians‐University of Munich Munich Germany; ^20^ Institute for Medical Informatics, Statistics and Epidemiology University of Leipzig Leipzig Germany; ^21^ LIFE‐Leipzig Research Centre for Civilization Diseases University of Leipzig Leipzig Germany

**Keywords:** Breast cancer predisposition, hereditary breast cancer

## Abstract

The prevalence of germ line mutations in non‐*BRCA1/2* genes associated with hereditary breast cancer (BC) is low, and the role of some of these genes in BC predisposition and pathogenesis is conflicting. In this study, 5589 consecutive BC index patients negative for pathogenic *BRCA1/2* mutations and 2189 female controls were screened for germ line mutations in eight cancer predisposition genes (*ATM*,*CDH1*,*CHEK2*,*NBN*,*PALB2*,*RAD51C*,*RAD51D,* and *TP53*). All patients met the inclusion criteria of the German Consortium for Hereditary Breast and Ovarian Cancer for germ line testing. The highest mutation prevalence was observed in the *CHEK2* gene (2.5%), followed by *ATM* (1.5%) and *PALB2* (1.2%). The mutation prevalence in each of the remaining genes was 0.3% or lower. Using Exome Aggregation Consortium control data, we confirm significant associations of heterozygous germ line mutations with BC for *ATM* (OR: 3.63, 95%CI: 2.67–4.94), *CDH1* (OR: 17.04, 95%CI: 3.54–82), *CHEK2* (OR: 2.93, 95%CI: 2.29–3.75), *PALB2* (OR: 9.53, 95%CI: 6.25–14.51), and *TP53* (OR: 7.30, 95%CI: 1.22–43.68). *NBN* germ line mutations were not significantly associated with BC risk (OR:1.39, 95%CI: 0.73–2.64). Due to their low mutation prevalence, the *RAD51C* and *RAD51D* genes require further investigation. Compared with control datasets, predicted damaging rare missense variants were significantly more prevalent in *CHEK2* and *TP53* in BC index patients. Compared with the overall sample, only *TP53* mutation carriers show a significantly younger age at first BC diagnosis. We demonstrate a significant association of deleterious variants in the *CHEK2*,*PALB2,* and *TP53* genes with bilateral BC. Both, *ATM* and *CHEK2*, were negatively associated with triple‐negative breast cancer (TNBC) and estrogen receptor (ER)‐negative tumor phenotypes. A particularly high *CHEK2* mutation prevalence (5.2%) was observed in patients with human epidermal growth factor receptor 2 (HER2)‐positive tumors.

## Introduction

The prevalence of heterozygous *BRCA1* and *BRCA2* germ line mutations and their associated risks for breast cancer (BC) and ovarian cancer (OC) have been extensively studied 1. Pathogenic *BRCA1* and *BRCA2* germ line mutations were found in approximately 17% of the index patients with BC who met the inclusion criteria of the German Consortium for Hereditary Breast and Ovarian Cancer (GC‐HBOC) for germ line testing 2. With the advent of next‐generation sequencing (NGS), germ line testing for hereditary BC/OC could be extended beyond the analysis of the *BRCA1* and *BRCA2* genes 3. However, gene panel sequencing and the testing of a number of potential risk genes are challenging, as international guidelines for the clinical management of patients carrying mutations in non‐*BRCA1/2* genes do not exist. Established non‐*BRCA1/2,* BC/OC risk genes are rarely mutated 4, 5, 6, 7, and data regarding their contribution to BC/OC risk are often controversial. Hence, verification is needed before the non‐*BRCA1/2* genes generally included in gene panel testing can be treated as confirmed BC/OC risk genes 8, 9. Moreover, criteria that predict mutation probabilities in non‐*BRCA1/2* genes are largely unknown and may differ from those that predict mutation probabilities in *BRCA1* and *BRCA2*.

The GC‐HBOC established multi‐gene panel testing in 2015. In addition to *BRCA1* and *BRCA2*, eight genes were defined as “core genes” based on data available at the time of the gene panel design, that suggested their association with BC (*ATM*,* CDH1*,* CHEK2*,* NBN*,* PALB2, and TP53*) or OC (*RAD51C and RAD51D*) 10, 11, 12, 13. With a relative risk of 5.3 (90% CI: 3.0–9.4), deleterious mutations in the *PALB2* gene appeared to confer high BC risk 14, 15, 16, 17. Lower relative risks were reported for mutations in the *CHEK2* (3.0, 90% CI: 2.6–3.5) 18, 19 and *ATM* genes (2.8, 90% CI: 2.2–3.7) 3, 20, 21, 22, 23. The *NBN* gene was considered as a BC predisposition gene mainly based on the genotyping results of a common founder mutation, c.657_661del, p.(Lys219Asnfs*16). For this variant, a meta‐analysis of 10 studies revealed a pooled OR of 2.66 (95% CI: 1.82–3.90; *P* < .001) 24. Mutations in *TP53* and *CDH1* are associated with multiple cancer types, one of which is BC. For *CDH1* mutations, a relative BC risk of 6.6 (90% CI: 2.2–19.9; *P* = .004) was reported 25, whereas reliable risk estimates for *TP53* mutations are missing. Mutations in the *RAD51C* and *RAD51D* genes have shown clear evidence of an association with OC, whereas evidence of an association with BC is limited 10, 26, 27. The aim of this study was to investigate the associations of germ line mutations in selected non‐*BRCA1/2* genes with BC risk and BC phenotype in a sample of 5589 *BRCA1/2* negative BC index patients who were recruited and counseled at university hospital‐based centers of the GC‐HBOC.

## Patients and Methods

### Patient sample

All patients met the inclusion criteria of the GC‐HBOC for germ line testing (Table S1). Of note, the GC‐HBOC inclusion criteria are not restricted to familial cases and also consider patients with early‐onset BC (age at first diagnosis [AAD] before 36 years), bilateral BC (AAD before 51 years), and patients affected by BC and OC even in the absence of a family history of BC and OC. In the absence of a patient with OC in a family, the available BC patient with the youngest AAD was defined as the index patient. In families with OC, a BC patient was defined as the index patient only when DNA derived from the OC patient was not available for genetic testing. Overall, 5589 female index patients with BC and without a personal history of OC were included in this study. All patients were counseled at a participating GC‐HBOC center. Physicians qualified in genetic counseling recorded personal and family BC/OC history, information regarding age at first BC diagnosis and tumor receptor status. Written informed consent was obtained from all patients, and ethical approval was granted by the ethics committee of the University of Cologne (07‐048). All patients were tested negative for pathogenic germ line variants in the *BRCA1* and *BRCA2* genes, including large genomic rearrangements (LGRs).

### Control sample

Two publicly accessible control datasets (ExAC and FLOSSIES) and sequencing data from 2189 geographically matched female controls were used in this study (Table 1). From the Exome Aggregation Consortium (ExAC) 28, we requested a dataset of individuals of European, non‐Finnish ancestry, excluding samples from The Cancer Genome Atlas (TCGA). This dataset comprises a total of 27,173 samples, which were analyzed by whole exome sequencing. The FLOSSIES project provides a dataset of 7325 women of European American ancestry (https://whi.color.com). All participating women have remained cancer‐free until at least 70 years of age. Germ line DNA samples of all participants were screened for variants in 27 established or suggested BC predisposition genes, including the eight selected non‐*BRCA1/2* genes. In addition, we sequenced germ line DNA samples of 2189 female control individuals of German descent (geographically matched controls; GMCs) by NGS and analyzed these samples for variants in the eight selected non‐*BRCA1/2* genes. Healthy controls were recruited by a study on genetic factors of a noncancer, age‐related phenotype, and a study on civilization diseases. The studies were approved by the local ethic committees, and all participants gave their written informed consent. At the time of blood draw, all GMCs were at least 40 years old (mean age 63, range 40–92) and cancer‐free.

**Table 1:** Clinical and BC tumor characteristics observed in the study sample (*n *=* *5589) and information on control reference groups (Geographically matched controls, GMCs; FLOSSIES, Female controls of European American ancestry, older than age 70 years and cancer‐free; ExAC, Non‐Finnish Europeans from the Exome Aggregation Consortium, excluding TCGA data) used in this study

	Number	%
BC index patients, overall	5589	100.0
Unilateral BC	4960	88.7
Bilateral BC	629	11.3
Without OC family history	4655	83.3
With OC family history	934	16.7
BC index patients, age at first BC diagnosis^a^	5589	100.0
<40	1440	25.8
40–49	2140	38.3
50–59	1269	22.7
≥60	691	12.4
Data not available	49	0.9
BC index patients, ER/PR/HER2 status available	3104	100.0
ER‐positive	2355	75.9
ER‐negative	749	24.1
PR‐positive	2188	70.5
PR‐negative	916	29.5
HER2‐positive	657	21.2
HER2‐negative	2447	78.8
TNBC	482	15.5
Non‐TNBC	2622	84.5
GMCs, age at blood draw	2189	100
40–49	147	6.7
50–59	324	14.8
≥60	1719	78.5
FLOSSIES	7325	100.0
ExAC	27,173	100.0

Abbreviations: ER, estrogen receptor; PR, progesterone receptor; HER2, human epidermal growth factor receptor 2; TNBC, triple‐negative breast cancer.

a The age at first diagnosis (AAD) was available for 5540 of 5589 BC index patients (mean AAD 46.7 years, range 17–92 years).

### Gene panel analysis

Genomic DNA was isolated from venous blood samples. NGS and data analysis were carried out at each participating center using Illumina sequencing platforms, employing either the customized TruRisk^®^ (Agilent or Illumina), a customized HaloPlex (Agilent, Santa Clara, California, USA), or the TruSight^™^ Cancer Sequencing Panel (Illumina, San Diego, California, USA) for target enrichment. All gene panels covered the eight selected non‐*BRCA1/2* core genes. The diagnostic pipelines of the labs involved have been successfully tested in European Molecular Genetics Quality Network (EMQN) schemes. Since LGRs cannot be detected reliably on the basis of NGS‐data 29, this mutation type was not included in this study. All deleterious variants affecting canonical non‐*BRCA1/2* core gene transcripts (*ATM,* NM_000051.3; *CDH1,* NM_004360.3; *CHEK2,* NM_007194; *NBN*, NM_002485.4; *PALB2,* NM_024675.3; *RAD51C,* NM_058216.2; *RAD51D*, NM_002878.3; *TP53,* NM_000546.5) were routinely verified by Sanger sequencing.

### Variant classification

Variant classification was performed in accordance with the regulations of the international ENIGMA consortium 30 (Evidence‐based Network for the Interpretation of Germ line Mutant Alleles; https://enigmaconsortium.org; version 1.1: 26 March 2015). All genetic variants were classified using a five‐tier variant classification system as proposed by the Unclassified Genetic Variants Working Group of the International Agency for Research on Cancer (IARC) (deleterious = class 5, likely deleterious = class 4, variant of uncertain significance (VUS) = class 3, likely benign = class 2, and benign = class 1) 31. According to the ENIGMA regulations 30, variants reported with a minor allele frequency (MAF) ≥1% in control reference groups (e.g., ExAC excluding TCGA, FLOSSIES) were generally considered benign (class 1). For simplification, class 4/5 variants were defined as “deleterious variants.” To investigate the associations of germ line mutations in selected non‐*BRCA1/2* genes with BC risk, only protein truncating variants (PTVs) were considered in patients and controls. PTVs were defined as nonsense, frameshift, or essential splice‐site variants affecting the invariant splice sites or the last nucleotide of an exon. As suggested by Lilyquist et al., protein truncating variants in the last exon or within the last 55 bp of the penultimate exon were classified as VUS, unless a known functional domain was disrupted 32. For the identification of potentially damaging, rare missense variants we employed two in silico prediction tools (SIFT and MutationTaster). Missense variants were defined as potentially damaging when predicted deleterious by both tools (Alamut version 2.10; Interactive Biosoftware, Rouen, France ) as of 30 November 2017.

### Statistical analysis

We performed case–control analyses for the investigation of the association of variants with the BC phenotype and case–case analyses for comparison of molecular subgroups. Univariate logistic regression was performed to estimate odds ratios (OR) and 95% confidence intervals (95%CI) using SPSS Statistics, Version 25 (IBM, Armonk, New York, USA). Fisher's exact test and the Student's *t*‐test (for age‐related analysis) were used to calculate levels of significance, with *P*‐values <0.05 considered significant.

## Results

### Associations of protein truncating variants in the selected core genes with BC

Detailed information on the patient sample is given in Table 1. Only protein truncating variants (PTVs) were considered to investigate the associations of germ line mutations in selected non‐*BRCA1/2* genes with BC risk in patients and controls. A list of all PTVs identified in the study sample is provided in Table S2. Among 5589 index patients with BC, 274 patients (4.9%) carried PTVs in the selected non‐*BRCA1/2* genes. The overall occurrence of PTVs in the selected core genes was markedly lower in all three control datasets compared with BC index patients. In the ExAC control dataset, 389 out of 27,173 individuals (1.4%) carried PTVs, which was comparable to the hypernormal control datasets FLOSSIES (67 of 7325 women, 0.9%) and GMCs (33 of 2189 women, 1.5%). When comparing mutation prevalence in BC index patients with ExAC data on a gene‐specific level, significant associations were observed for *ATM* (OR: 3.63, 95% CI 2.67–4.94; *P* < .0001), *CHEK2* (OR: 2.93, 95% CI 2.29–3.75; *P* < .0001), *PALB2* (OR: 9.53, 95% CI 6.25–14.51; *P* < .0001), *CDH1* (OR: 17.04, 95% CI 3.54–82; *P* < .0001), and *TP53* (OR: 7.30, 95% CI 1.22–43.68; *P* = .038) (Table 2). In contrast, we did not observe significant associations between PTVs in the *NBN* gene and BC (OR: 1.39, 95% CI 0.73–2.64; *P* = .363). When comparing the PTV prevalence in BC index patients with hypernormal controls (FLOSSIES, GMCs), higher ORs were observed for *ATM*,* CHEK2,* and *PALB2* than were observed in comparison with ExAC data (Table 2). Again, no association between PTVs in the *NBN* gene and BC was observed. In contrast to the aforementioned genes, the analysis of *RAD51C* and *RAD51D* revealed ambiguous results. For *RAD51C,* a significant association with BC was observed when comparing PTV prevalence with FLOSSIES but not when compared with ExAC data or GMCs (Table 2). For *RAD51D*, ORs of 3.04 (vs. ExAC data) and 3.28 (vs. FLOSSIES) were observed, though both comparisons did not reach levels of significance (Table 2).

**Table 2:** Prevalence of protein truncating variants (PTVs) in eight non‐*BRCA1/2* cancer predisposition genes in 5589 BC index patients compared with control datasets (ExAC, FLOSSIES, and GMCs). Percentages of individuals carrying a mutation in the respective datasets are shown in parentheses

Gene	BC *n* = 5589 (%)	ExAC *n* = 27,173 (%)	FLOSSIES *n* = 7325 (%)	GMCs *n* = 2189 (%)	BC vs. ExAC OR (95% CI, *P* a)	BC vs. FLOSSIES OR (95% CI, *P* a)	BC vs. GMCs OR (95% CI, *P* a)
*ATM*	71 (1.27)	96 (0.35)	14 (0.19)	9 (0.41)	3.63 (2.67–4.94, <0.0001)	6.72 (3.78–11.93, <0.0001)	3.12 (1.56–6.25, 0.0004)
*CDH1*	7 (0.13)	2 (0.01)	0 (0.0)	0 (0.0)	17.04 (3.54–82, <0.0001)	n.a. (n.a., 0.0028)	n.a. (n.a., 0.2020)
*CHEK2*	103 (1.84)	172 (0.63)	28 (0.38)	11 (0.50)	2.93 (2.29–3.75, <0.0001)	4.87 (3.20–7.40, <0.0001)	3.72 (1.99–6–94, <0.0001)
*CHEK2*, c.1100del	79 (1.41)	127 (0.47)	22 (0.30)	8 (0.37)	3.02 (2.28–4.01, <0.0001)	4.71 (2.93–7.56, <0.0001)	3.91 (1.87–8.05, <0.0001)
*NBN*	12 (0.21)	42 (0.15)	14 (0.19)	9 (0.41)	1.39 (0.73–2.64, 0.3630)	1.12 (0.53–2.43, 0.8438)	0.52 (0.22–1.24, 0.1466)
*PALB2*	64 (1.15)	33 (0.12)	7 (0.10)	2 (0.09)	9.53 (6.25–14.51, <0.0001)	12.11 (5.55–26.45 < 0.0001)	12.67 (3.10–51.79, <0.0001)
*RAD51C*	9 (0.16)	34 (0.13)	2 (0.03)	2 (0.09)	1.29 (0.62–2.69, 0.5409)	5.91 (1.28–27.34, 0.0129)	1.76 (0.38–8.17, 0.7384)
*RAD51D*	5 (0.09)	8 (0.03)	2 (0.03)	0 (0.0)	3.04 (0.99–9.30, 0.0558)	3.28 (0.64–16.91, 0.2512)	n.a. (n.a., 0.3308)
*TP53*	3 (0.05)	2 (0.01)	0 (0.0)	0 (0.0)	7.30 (1.22–43.68, 0.0378)	n.a. (n.a., 0.0810)	n.a. (n.a., 0.5640)
PTVs	274	389	67	33			
Carriers^b^	272 (4.87)	389 (1.43)	67 (0.91)	33 (1.51)			

OR, odds ratio; CI, confidence interval.

a Fisher's exact test.

b Two patients carried two PTVs (*ATM* and *CHEK2*;* NBN* and *RAD51C*).

### Deleterious variants in non‐*BRCA1/2* genes in the overall patient sample

Overall, heterozygous deleterious variants in at least one of the eight non‐*BRCA1/2* core genes were present in 339 of the 5589 BC index patients (6.1%, Table 3). The highest prevalence of deleterious variants was observed in the *CHEK2* gene (138 carriers, 2.5%), followed by *ATM* (81 carriers, 1.4%) and *PALB2* (68 carriers, 1.2%). The prevalence of deleterious variants in the *CDH1*,* NBN*,* RAD51C*,* RAD51D,* and *TP53* genes in the overall patient sample was 0.3% or lower for each gene (Table 3). Overall, 147 distinct deleterious variants were identified in 339 patients, of which 45 were recurrent (Table). With a carrier frequency of 1.4%, the c.1100del founder variant in the *CHEK2* gene was the most prevalent deleterious variant observed (Table 3).

**Table 3:** Deleterious variants in eight non‐*BRCA1/2* core genes identified in 5589 BC index patients. Total numbers of patients carrying deleterious variants in each gene are given. Percentages of patients carrying a deleterious variant in the respective subgroup are shown in parentheses

Gene	BC index patients			
	All, *n* = 5589 (%)	Bilateral BC, *n* = 629 (%)	Unilateral BC, *n* = 4960 (%)	Bilateral vs. unilateral BC OR (95% CI, *P* a)
*ATM*	81 (1.45)	10 (1.59)	71 (1.43)	1.11 (0.57–2.17, 0.7229)
*CDH1*	8 (0.14)	1 (0.16)	7 (0.14)	1.13 (0.14–9.17, 1.0000)
*CHEK2* a	138 (2.47)	20 (3.18)	118 (2.38)	1.35 (0.83–2.18, 0.2196)
*CHEK2,* c.1100del	79 (1.41)	**16 (2.54)**	**63 (1.27)**	2.03 (1.17–3.53, 0.0180)
*NBN*	12 (0.21)	0 (0.00)	12 (0.24)	n.a. (n.a., 0.3830)
*PALB2*	68 (1.22)	**14 (2.23)**	**54 (1.09)**	2.07 (1.14–3.75, 0.0201)
*RAD51C*	11 (0.20)	1 (0.16)	10 (0.20)	0.79 (0.10–6.17, 1.0000)
*RAD51D*	6 (0.11)	1 (0.16)	5 (0.10)	1.58 (0.18–13.53, 0.5116)
*TP53*	17 (0.30)	**5 (0.79)**	**12 (0.24)**	3.30 (1.16–9.41, 0.0348)
Deleterious variants	341	**52**	**289**	1.46 (1.07–1.98, 0.0211)
Carriers^b^	339 (6.07)	**52 (8.27)**	**287 (5.79)**	1.50 (1.10–2.04, 0.0124)

a The disease‐associated *CHEK2* variant c.470C>T, p.I157T was classified as a low‐risk variant for BC based on a recent meta‐analysis including 15,985 BC cases and 18,609 controls from eight studies (OR: 1.58, 95% CI 1.42–1.75; *P* < .00001)^37^. In our study, the c.470C>T, p.I157T was heterozygously present in 86 out of 5589 BC index patients (1.54%). In contrast, 44 heterozygous mutation carriers were identified among the 2189 hypernormal female controls of German descent analyzed in this study (2.0%). Due to the lack of association with BC in our sample, this variant was not considered to be a deleterious mutation in our study.

b Two patients carried two deleterious variants (*ATM* and *CHEK2*;* NBN* and *RAD51C*). Mutation prevalence showing significant differences according to the subgroups (bilateral BC vs. unilateral BC) are shown in bold. n.a. = not applicable.

### Deleterious variants in non‐*BRCA1/2* genes according to personal and family history of cancer

Among the 5589 BC index patients, 629 were affected by bilateral BC. In this subgroup, 8.3% of patients (52 of 629) carried a deleterious variant in at least one of the eight selected genes. This was significantly higher than the prevalence of deleterious variants in the 4960 BC index patients affected by unilateral BC (5.8%, 289 in 4960; *P* = .021). On a gene‐specific level, the *CHEK2* c.1000del founder variant (2.5% vs. 1.3%; *P* = .018), deleterious variants in the *PALB2* gene (2.2% vs. 1.1%; *P* = .020), and deleterious variants in the *TP53* gene (0.79% vs. 0.24%; *P* = .035) were significantly associated with bilateral BC (Table 3). Of the 5589 BC index patients, 934 reported a family history of OC. The prevalence of deleterious variants was not significantly different in BC index patients with a family history of OC versus the 4655 BC index patients who had no family history of OC (data not shown).

### Deleterious variants in non‐*BRCA1/2* genes according to age at first diagnosis of BC

In a gene‐specific analysis, only patients carrying deleterious *TP53* variants showed a younger mean age at first BC diagnosis (*TP53*: 39.7 years, range 23–71 years) compared with the overall sample (46.7 years, range 17–92 years), with differences reaching levels of significance (*P* = .004, Student's *t*‐test, Table 4). For example, 52.9% (9 of 17) of the *TP53* mutation carriers showed an age at first BC diagnosis below the age of 40 years compared with 26.0% (1440 of 5540) of the BC index patients overall (*P* = .021, Table 4). Overall, no significant difference in age at first BC diagnosis was observed between patients carrying deleterious variants and the overall sample (Table 4).

**Table 4:** Age at first diagnosis of BC in the overall sample and according to germ line mutation status. Included are patients carrying deleterious variants in the respective risk gene. Compared with the overall sample, only *TP53* mutation carriers showed a significantly younger age at first diagnosis

Gene	Age at first diagnosis						
	Total	Mean (years)	Median (years)	Range (years)	<40 years (% of carriers)	<50 years (% of carriers)	<60 years (% of carriers)
Overall^a^	5540	46.7	46	17–92	1440 (26.0)	3580 (64.6)	4849 (87.5)
*ATM*	81	45.0	45	27–80	28 (34.6)	58 (71.6)	71 (87.7)
*CDH1*	8	45.1	43	33–59	3 (37.5)	6 (75.0)	8 (100.0)
*CHEK2*	138	47.1	46	29–75	35 (25.4)	92 (66.7)	120 (87.0)
*NBN*	12	46.3	50	33–74	3 (25.0)	6 (50.0)	11 (91.7)
*PALB2*	68	45.8	46	28–78	20 (29.4)	44 (64.7)	62 (91.2)
*RAD51C*	11	44.9	48	33–53	4 (36.4)	6 (54.5)	11 (100.0)
*RAD51D*	6	46.8	49	33–60	1 (16.7)	3 (50.0)	5 (83.3)
*TP53*	17	39.7	35	23–71	9 (52.9)	14 (82.4)	16 (94.1)
All carriers	339	45.9	45	23–80	94 (27.7)	228 (67.3)	301 (88.8)

a The age at first diagnosis of BC was available for 5540 of 5589 BC index patients.

### Deleterious variants in BC index patients according to ER/PR/HER2 status

Information regarding hormone receptor (ER/PR) and HER2 status was available for a subgroup of 3104 of the 5589 BC index cases. Within this subgroup, 482 showed a triple‐negative breast cancer (TNBC) phenotype (15.5%, Table 1). The mutation prevalence in patients with ER‐positive BC was significantly increased in patients with ER‐negative tumors (7.3% vs. 4.7%, *P* = .014; Table 5). In a gene‐specific analysis, this difference was highest in the *ATM* (1.83% vs. 0.53%, *P* = .009; Table 5) and *CHEK2* genes (3.2% vs. 1.9%, *P* = .060; Table 5). The overall prevalence of deleterious variants was not significantly different when stratified by PR or HER2 status (Table 5). On a gene‐specific level, however, a particularly high *CHEK2* mutation prevalence was observed in patients with HER2‐positive tumors compared with patients with HER2‐negative tumors (5.2% vs. 2.3%; *P* < .001; Table 5). TNBC predicts a significantly lower mutation probability in the selected genes. While deleterious mutations were present in 186 of the 2622 patients with non‐TNBC (7.1%), only 20 of the 482 patients with TNBC (4.1%) carried a deleterious mutation (*P* = .017; Table 5). Both the *ATM* and *CHEK2* genes showed significantly higher mutation rates in patients with non‐TNBC versus patients with a TNBC tumor phenotype (*ATM*: 1.7% vs. 0.4%; *P* = .026; *CHEK2*: 3.3% vs. 0.8%, *P* = .002; Table 5).

**Table 5:** Deleterious variants in the eight selected non‐*BRCA1/2* core genes identified in 5589 BC index patients. Total numbers of patients carrying deleterious variants in each gene are given. Percentages of patients carrying a mutation in the respective subgroup are shown in parentheses. Mutation prevalence showing significant differences according to the subgroups are shown in bold (ER‐positive vs. ER‐negative; PR‐positive vs. PR‐negative; HER2‐positive vs. HER2‐negative; non‐TNBC vs. TNBC)

Gene	BC index patients								
	ER/PR/HER2 status available *n* = 3104 (%)	ER‐positive *n* = 2355 (%)	ER‐negative *n* = 749 (%)	PR‐positive *n* = 2188 (%)	PR‐negative *n* = 916 (%)	HER2‐positive *n* = 657 (%)	HER2‐negative *n* = 2447 (%)	Non‐TNBC *n* = 2622 (%)	TNBC *n* = 482 (%)
*ATM*	47 (1.51)	**43 (1.83)**	**4 (0.53)**	39 (1.78)	8 (0.87)	7 (1.07)	40 (1.63)	**45 (1.72)**	**2 (0.41)**
*CDH1*	7 (0.23)	6 (0.25)	1 (0.13)	4 (0.18)	3 (0.33)	1 (0.15)	6 (0.25)	6 (0.23)	1 (0.21)
*CHEK2*	90 (2.90)	76 (3.23)	14 (1.87)	70 (3.20)	20 (2.18)	**34 (5.18)**	**56 (2.29)**	**86 (3.28)**	**4 (0.83)**
*CHEK2,* c.1100del	52 (1.68)	43 (1.83)	9 (1.20)	42 (1.92)	10 (1.09)	**20 (3.04)**	**32 (1.31)**	49 (1.87)	3 (0.62)
*NBN*	5 (0.16)	4 (0.17)	1 (0.13)	3 (0.14)	2 (0.22)	0 (0.00)	5 (0.20)	4 (0.15)	1 (0.21)
*PALB2*	40 (1.29)	31 (1.32)	9 (1.20)	29 (1.33)	11 (1.20)	4 (0.61)	36 (1.47)	32 (1.22)	8 (1.66)
*RAD51C*	4 (0.13)	3 (0.13)	1 (0.13)	2 (0.09)	2 (0.22)	1 (0.15)	3 (0.12)	3 (0.11)	1 (0.21)
*RAD51D*	3 (0.10)	1 (0.04)	2 (0.27)	1 (0.05)	2 (0.22)	0 (0.00)	3 (0.12)	1 (0.04)	2 (0.41)
*TP53*	10 (0.32)	7 (0.30)	3 (0.40)	8 (0.37)	2 (0.22)	5 (0.76)	5 (0.20)	9 (0.34)	1 (0.21)
Carriers	**206 (6.64)**	**171 (7.26)**	**35 (4.67)**	**156 (7.13)**	**50 (5.46)**	**52 (7.91)**	**154 (6.29)**	**186 (7.09)**	**20 (4.15)**

### Variants of uncertain significance (VUS) and rare missense variants in the overall patient sample

In 827 of the 5589 index patients (14.8%; Table S2), we identified 421 distinct variants of uncertain significance (VUS). Most VUS were missense variants (396 of 421; 94.1%). Most VUS were present in the *ATM* gene (affecting 322 of 5589 patients; 5.8%), followed by the *CHEK2* (196 of 5589 patients; 3.5%) and *PALB2* genes (76 of 5589 patients; 1.4%). The vast majority of patients with an uncertain genetic report carry a single VUS in the selected genes (761 of 829; 91.8%). To examine the potential association of missense variants in the 8 investigated genes with BC risk, we next focused on rare missense variants (MAF < 0.1%), which were predicted to be damaging by both, SIFT and MutationTaster (Table S3). Compared with ExAC controls, rare *CHEK2* and *TP53* missense variants predicted damaging by both tools were significantly more prevalent in BC patients (1.43% vs. 0.71%; *P* < .0001 and 0.41% vs. 0.18%; *P* = .002). Prevalence of these rare missense variants in *ATM*,* PALB2*,* RAD51C,* and *RAD51D* was only marginally increased in comparison with ExAC controls, which differences not reaching levels of statistical significance.

## Discussion

In our sample of 5589 BC index cases, we confirmed that PTVs in the *ATM*,* CDH1*,* CHEK2*,* PALB2,* and *TP53* genes increase BC risk. The ORs for the most frequently mutated genes (*ATM* (OR: 3.63, 95% CI 2.67–4.94), *CHEK2* (OR: 2.93, 95% CI 2.29–3.75), and *PALB2* (OR: 9.53, 95% CI 6.25–14.51) are compatible with published data, according to a meta‐analysis focusing on PTVs 33. In this meta‐analysis, an aggregated OR of 3.20 (95% CI 2.04–5.04; analysis of 4266 cases and 5566 controls) was calculated for *ATM*, an aggregated OR of 3.25 (95% CI 2.55–4.13, analysis of 7263 cases and 13,785 controls) was calculated for *CHEK2*, and an aggregated OR of 21.40 (95% CI 10.10–45.32, analysis of 5862 cases and 17,453 controls) was calculated for *PALB2*. Based on a recent analysis of a large series of BC patients of European ancestry, Couch et al. reported ORs of 3.13 (95% CI 2.33–4.23) for *ATM*, 2.23 (95% CI 1.85–2.69) for *CHEK2,* and 7.67 (95% CI 5.19–11.50) for *PALB2* when focusing on PTVs 4. Similar ORs were also observed for these genes in the Australian population 9. For the syndrome‐associated genes *CDH1* and *TP53*, we confirm significant associations with BC, though with wide confidence intervals.

In contrast to previous studies focusing on the c.657_661del, p.(Lys219Asnfs*16) founder mutation 24, we could not confirm *NBN* as a BC predisposition gene (OR: 1.39, 95% CI 0.73–2.64; *P* = .363; Table 2). This is consistent with the panel gene analyses by Couch et al. (OR: 1.27, 95% CI 0.81–2.01; *P* = .32) and Thompson et al. (OR: 0.67, 95% CI 0.11–4.0; *P* = 1.00) 4, 9. For *RAD51D* mutations, we demonstrated some indication of an association with BC (OR: 3.04, 95% CI 0.99–9.30; *P* = .0558), which is compatible with the results obtained by Couch et al. using the same control dataset (OR: 2.90, 95% CI 1.12–7.21; *P* = .02) 4. However, these associations did not reach levels of significance. Thus, we suggest that larger collaborative studies are necessary to assess the role of *RAD51D* in BC pathogenesis. The same holds true for *RAD51C* mutations for which we showed an elevated prevalence in BC index patients versus hypernormal controls but not versus ExAC data (Table 2).

We demonstrated significantly higher mutation prevalence in bilateral versus unilateral BC cases, with highest differences in *CHEK2*,* PALB2,* and *TP53* (Table 3). In agreement with the data presented here, Couch et al. showed that pathogenic variants in *CHEK2, PALB2*, and *TP53* were associated with bilateral BC 4. A young age at BC disease onset, a personal or family history of OC, and the occurrence of the TNBC tumor phenotype predict high mutation probabilities in the *BRCA1* gene and to a lesser extent in the *BRCA2* gene 2. However, these criteria do not effectively enrich for patients with mutations in non‐*BRCA1/2* genes. In this investigation, age at first BC diagnosis did not significantly predict mutation probabilities overall, with the exception of *TP53* mutations, which is well in line with published data 34, 35. The mutation prevalence in non‐*BRCA1/2* genes stratified by age likewise did not differ markedly in the studies of Thompson et al. and Buys et al. 5, 9. Of note, the subgroup of patients with TNBC, a tumor phenotype closely associated with a high *BRCA1* mutation prevalence 35, 36, showed lower mutation probabilities in non‐*BRCA1/2* genes, especially for *ATM* and *CHEK2* mutations, we identified a negative association with this subtype (Table 5). Similar results were observed by Buys et al., demonstrating significantly lower mutation probabilities for both genes in patients with TNBC versus other subtypes 5. Deleterious *ATM* and *CHEK2* mutations were particularly frequent in ER‐positive tumors, while *CHEK2* mutations were also frequently found in HER2‐positive tumors.

Our study confirmed the benefit of multi‐gene testing for risk assessment in BC/OC families. Here, we identified deleterious variants in validated BC predisposition genes (*ATM*,* CDH1*,* CHEK2*,* PALB2*, and *TP53*) in 312 of 5589 BC index cases, enabling the offer of predictive testing and adjusted surveillance programs in these families. Of note, we identified a high prevalence of VUS which is still a major drawback of multi‐gene testing in a diagnostic setting.

## Conflict of Interest

The authors have nothing to disclose.

## Supporting information


**Table S1.** Inclusion criteria for germline testing.
**Table S2.** Class 3, 4 and 5 mutations identified in the study sample.
**Table S3.** Prevalence of rare missense variants.
