# Supplementary material for: Gene panel testing of 5589 BRCA1/2‐negative index patients with breast cancer in a routine diagnostic setting: results of the German Consortium for Hereditary Breast and Ovarian Cancer
Source: Cancer Med. 2018 Mar 9;7(4):1349–58. doi: 10.1002/cam4.1376 (PMC5911592; doi:10.1002/cam4.1376)
Supplement: Supplementary file 1 — Table S1. Inclusion criteria for germline testing. Table S2. Class 3, 4 and 5 mutations identified in the study sample. Table S3. Prevalence of rare missense variants. [file CAM4-7-1349-s001.docx]

**Gene panel testing of 5,589 *BRCA1/2*-negative index patients with breast cancer in a routine diagnostic setting: Results of the German Consortium for Hereditary Breast and Ovarian Cancer**

On-line Only Supplemental Material:

**Table S1:** Inclusion criteria for germline testing

**Table S2:** Class 3, 4 and 5 mutations identified in the study sample

**Table S3:** Prevalence of rare missense variants

- ≥3 women with breast cancer
- ≥2 women with breast cancer, 1 with onset below 51 years of age
- ≥1 woman with breast cancer and 1 woman with ovarian cancer
- ≥2 women with ovarian cancer
- ≥1 woman with breast- and ovarian cancer
- ≥1 woman with breast cancer below 36 years of age
- ≥1 woman with bilateral breast cancer with onset below 51 years
- ≥1 male with breast cancer and 1 woman with breast- or ovarian cancer

**Table S1:** Inclusion criteria of the German Consortium for Hereditary Breast and Ovarian Cancer (GC-HBOC) for *BRCA1* and *BRCA2* germline testing. Ductal carcinoma in situ (DCIS) is categorized as breast cancer.

| **gene** | **NCBI accession** | **genomic coordinates** | **exon/intron** | **HGVS (nucleotide)** | **HGVS (protein)** | **HGMD** | **classification** | **consequence** | **no. of patients** | **category** |
| --- | --- | --- | --- | --- | --- | --- | --- | --- | --- | --- |
| *ATM* | NM_000051.3 | Chr11(GRCh37):g.108098352A>G | 2/63 | c.1A>G | p.(Met1?) | CM122039 | Class4 | start lost | 1 | PTV |
| *ATM* | NM_000051.3 | Chr11(GRCh37):g.108098354G>A | 2/63 | c.3G>A | p.(Met1?) | CM030187 | Class4 | start lost | 1 | PTV |
| *ATM* | NM_000051.3 | Chr11(GRCh37):g.108106422del | 5/63 | c.356del | p.(Glu119Aspfs*3) | / | Class5 | frameshift | 1 | PTV |
| *ATM* | NM_000051.3 | Chr11(GRCh37):g.108106515_108106518del | 5/63 | c.450_453del | p.(Ser151*) | CD067145 | Class5 | nonsense | 1 | PTV |
| *ATM* | NM_000051.3 | Chr11(GRCh37):g.108114679G>T | intron5 | c.497-1G>T | p.(?) | / | Class4 | splice defect | 1 | PTV |
| *ATM* | NM_000051.3 | Chr11(GRCh37):g.108114721C>T | 6/63 | c.538C>T | p.(Gln180*) | CM068641 | Class5 | nonsense | 1 | PTV |
| *ATM* | NM_000051.3 | Chr11(GRCh37):g.108115533dup | 7/63 | c.681dup | p.(Gly228Argfs*26) | / | Class5 | frameshift | 1 | PTV |
| *ATM* | NM_000051.3 | Chr11(GRCh37):g.108115642del | 7/63 | c.790del | p.(Tyr264Ilefs*12) | CD991594 | Class5 | frameshift | 2 | PTV |
| *ATM* | NM_000051.3 | Chr11(GRCh37):g.108115731del | 7/63 | c.879delA | p.(Gly294Glufs*26) | / | Class5 | frameshift | 1 | PTV |
| *ATM* | NM_000051.3 | Chr11(GRCh37):g.108119806_108119807del | 9/63 | c.1212_1213del | p.(Asn405*) | CD1613093 | Class5 | nonsense | 1 | PTV |
| *ATM* | NM_000051.3 | Chr11(GRCh37):g.108121477_108121480del | 10/63 | c.1285_1288del | p.(Asn429Valfs*7) | / | Class5 | frameshift | 1 | PTV |
| *ATM* | NM_000051.3 | Chr11(GRCh37):g.108121756_108121757del | 10/63 | c.1564_1565del | p.(Glu522Ilefs*43) | CD961794 | Class5 | frameshift | 3 | PTV |
| *ATM* | NM_000051.3 | Chr11(GRCh37):g.108122616del | 11/63 | c.1660del | p.(Thr554Argfs*2) | CD087217 | Class5 | frameshift | 2 | PTV |
| *ATM* | NM_000051.3 | Chr11(GRCh37):g.108122674del | 11/63 | c.1718del | p.(Lys573Argfs*4) | / | Class5 | frameshift | 1 | PTV |
| *ATM* | NM_000051.3 | Chr11(GRCh37):g.108122693G>A | 11/63 | c.1737G>A | p.(Trp579*) | / | Class5 | nonsense | 2 | PTV |
| *ATM* | NM_000051.3 | Chr11(GRCh37):g.108123641T>G | intron12 | c.1898+2T>G | p.(?) | CS982068 | Class4 | splice defect | 1 | PTV |
| *ATM* | NM_000051.3 | Chr11(GRCh37):g.108127067G>A | 14/63 | c.2250G>A | p.(=) | CS982070 | Class4 | splice defect, last nt. exon 14 | 1 | PTV |
| *ATM* | NM_000051.3 | Chr11(GRCh37):g.108129803del | intron16 | c.2466+1del | p.(?) | CD961798 | Class4 | splice defect | 1 | PTV |
| *ATM* | NM_000051.3 | Chr11(GRCh37):g.108137928G>T | 17/63 | c.2497G>T | p.(Gly833*) | / | Class5 | nonsense | 1 | PTV |
| *ATM* | NM_000051.3 | Chr11(GRCh37):g.108151895G>A | 24/63 | c.3576G>A | p.(?) | CS961477 | Class5 | splice defect, last nt. exon 24 | 1 | PTV |
| *ATM* | NM_000051.3 | Chr11(GRCh37):g.108153572_108153576del | 25/63 | c.3712_3716del | p.(Leu1238Lysfs*6) | CD961799 | Class5 | frameshift | 2 | PTV |
| *ATM* | NM_000051.3 | Chr11(GRCh37):g.108155009del | 26/63 | c.3802del | p.(Val1268*) | CD961800 | Class5 | nonsense | 3 | PTV |
| *ATM* | NM_000051.3 | Chr11(GRCh37):g.108155059del | 26/63 | c.3852del | p.(Asp1285Thrfs*8) | / | Class5 | frameshift | 1 | PTV |
| *ATM* | NM_000051.3 | Chr11(GRCh37):g.108155098T>G | 26/63 | c.3891T>G | p.(Tyr1297*) | / | Class5 | nonsense | 1 | PTV |
| *ATM* | NM_000051.3 | Chr11(GRCh37):g.108159737dup | 28/63 | c.4143dup | p.(Pro1382Serfs*6) | CI991966 | Class5 | frameshift | 1 | PTV |
| *ATM* | NM_000051.3 | Chr11(GRCh37):g.108163496T>A | 30/63 | c.4587T>A | p.(Tyr1529*) | / | Class5 | nonsense | 1 | PTV |
| *ATM* | NM_000051.3 | Chr11(GRCh37):g.108164169dup | 31/63 | c.4741dupA | p.(Ile1581Asnfs*5) | / | Class5 | frameshift | 2 | PTV |
| *ATM* | NM_000051.3 | Chr11(GRCh37):g.108168069del | 33/63 | c.4965del | p.(Lys1656Argfs*4) | / | Class5 | frameshift | 1 | PTV |
| *ATM* | NM_000051.3 | Chr11(GRCh37):g.108170613G>A | intron34 | c.5177+1G>A | p.(?) | CS083866 | Class5 | splice defect | 1 | PTV |
| *ATM* | NM_000051.3 | Chr11(GRCh37):g.108172364_108172383del | intron34 | c.5178-11_5186del | p.(?) | / | Class4 | splice defect | 1 | PTV |
| *ATM* | NM_000051.3 | Chr11(GRCh37):g.108172518T>C | intron35 | c.5319+2T>C | p.(?) | CS067074 | Class5 | splice defect | 1 | PTV |
| *ATM* | NM_000051.3 | Chr11(GRCh37):g.108175400A>C | intron36 | c.5497-2A>C | p.(?) | CS991306 | Class4 | splice defect | 2 | PTV |
| *ATM* | NM_000051.3 | Chr11(GRCh37):g.108178641C>T | 38/63 | c.5692C>T | p.(Arg1898*) | CM066736 | Class5 | nonsense | 1 | PTV |
| *ATM* | NM_000051.3 | Chr11(GRCh37):g.108183151G>T | 40/63 | c.5932G>T | p.(Glu1978*) | CM980147 | Class5 | nonsense | 3 | PTV |
| *ATM* | NM_000051.3 | Chr11(GRCh37):g.108183223C>T | 40/63 | c.6004C>T | p.(Gln2002*) | CM044575 | Class4 | nonsense | 1 | PTV |
| *ATM* | NM_000051.3 | Chr11(GRCh37):g.108186638G>A | 41/63 | c.6095G>A | p.(Arg2032Lys) | CM990215 | Class5 | splice defect, last nt. exon 41 | 1 | PTV |
| *ATM* | NM_000051.3 | Chr11(GRCh37):g.108186742C>T | 42/63 | c.6100C>T | p.(Arg2034*) | CM960102 | Class5 | nonsense | 1 | PTV |
| *ATM* | NM_000051.3 | Chr11(GRCh37):g.108188106C>T | 43/63 | c.6205C>T | p.(Gln2069*) | / | Class5 | nonsense | 1 | PTV |
| *ATM* | NM_000051.3 | Chr11(GRCh37):g.108196893_108196894del | 47/63 | c.6916_6917del | p.(Leu2307Cysfs*65) | CD982463 | Class5 | frameshift | 2 | PTV |
| *ATM* | NM_000051.3 | Chr11(GRCh37):g.108200960C>T | 50/63 | c.7327C>T | p.(Arg2443*) | CM960104 | Class5 | nonsense | 2 | PTV |
| *ATM* | NM_000051.3 | Chr11(GRCh37):g.108202172_108202175del | 51/63 | c.7517_7520del | p.(Arg2506Thrfs*3) | CD961811 | Class5 | frameshift | 1 | PTV |
| *ATM* | NM_000051.3 | Chr11(GRCh37):g.108202218C>G | 51/63 | c.7563C>G | p.(Tyr2521*) | / | Class5 | nonsense | 1 | PTV |
| *ATM* | NM_000051.3 | Chr11(GRCh37):g.108202604A>C | intron51 | c.7630-2A>C | p.(?) | CS995311 | Class4 | splice defect | 5 | PTV |
| *ATM* | NM_000051.3 | Chr11(GRCh37):g.108205783A>T | 55/63 | c.8098A>T | p.(Lys2700*) |  | Class5 | nonsense | 1 | PTV |
| *ATM* | NM_000051.3 | Chr11(GRCh37):g.108206684dup | 56/63 | c.8264dup | p.(Tyr2755*) | CI122170 | Class5 | nonsense | 1 | PTV |
| *ATM* | NM_000051.3 | Chr11(GRCh37):g.108214075_108214084del | 57/63 | c.8395_8404del | p.(Phe2799Lysfs*4) | CD982468 | Class5 | frameshift | 1 | PTV |
| *ATM* | NM_000051.3 | Chr11(GRCh37):g.108216600T>A | 58/63 | c.8549T>A | p.(Leu2850*) | / | Class5 | nonsense | 2 | PTV |
| *ATM* | NM_000051.3 | Chr11(GRCh37):g.108216637T>C | intron58 | c.8584+2T>C | p.(?) | / | Class4 | splice defect | 2 | PTV |
| *ATM* | NM_000051.3 | Chr11(GRCh37):g.108224587dup | 60/63 | c.8766dup | p.(Val2923Cysfs*2) | CI983037 | Class5 | frameshift | 1 | PTV |
| *ATM* | NM_000051.3 | Chr11(GRCh37):g.108236071_108236098del | 63/63 | c.9007_9034del | p.(Asn3003Aspfs*6) | / | Class5 | frameshift, affecting kinase domain | 1 | PTV |
| *ATM* | NM_000051.3 | Chr11(GRCh37):g.108236083G>T | 63/63 | c.9019G>T | p.(Glu3007*) | CM980162 | Class5 | nonsense, affecting kinase domain | 1 | PTV |
| *CDH1* | NM_004360.3 | Chr16(GRCh37):g.68772211G>A | 2/16 | c.60G>A | p.(Trp20*) | / | Class5 | nonsense | 1 | PTV |
| **gene** | **NCBI accession** | **genomic coordinates** | **exon/intron** | **HGVS (nucleotide)** | **HGVS (protein)** | **HGMD** | **classification** | **consequence** | **no. of patients** | **category** |
| *CDH1* | NM_004360.3 | Chr16(GRCh37):g.68772237del | 2/16 | c.86del | p.(His29Profs*27) | / | Class5 | frameshift | 1 | PTV |
| *CDH1* | NM_004360.3 | Chr16(GRCh37):g.68842667del | 5/16 | c.603del | p.(Val202Leufs*13) | / | Class5 | frameshift | 1 | PTV |
| *CDH1* | NM_004360.3 | Chr16(GRCh37):g.68846166G>A | 8/16 | c.1137G>A | p.(=) | CS060517 | Class4 | splice defect, last nt. exon 8 | 1 | PTV |
| *CDH1* | NM_004360.3 | Chr16(GRCh37):g.68853204del | 11/16 | c.1587del | p.(Ala530Profs*27) | / | Class5 | frameshift | 2 | PTV |
| *CDH1* | NM_004360.3 | Chr16(GRCh37):g.68857329del | 13/16 | c.1964del | p.(Lys655Argfs*4) | / | Class5 | frameshift | 1 | PTV |
| *CHEK2* | NM_007194.3 | Chr22(GRCh37):g.29130642del | 2/15 | c.68delG | p.(Gly23Alafs*38) | / | Class5 | frameshift | 1 | PTV |
| *CHEK2* | NM_007194.3 | Chr22(GRCh37):g.29130494A>C | 2/15 | c.216T>G | p.(Tyr72*) | / | Class5 | nonsense | 1 | PTV |
| *CHEK2* | NM_007194.3 | Chr22(GRCh37):g.29121266G>A | 3/15 | c.409C>T | p.(Arg137*) | / | Class5 | nonsense | 1 | PTV |
| *CHEK2* | NM_007194.3 | Chr22(GRCh37):g.29121230C>T | intron3 | c.444+1G>A | p.(?) | CS030536 | Class4 | splice defect | 13 | PTV |
| *CHEK2* | NM_007194.3 | Chr22(GRCh37):g.29121050del | 4/15 | c.507del | p.(Phe169Leufs*2) | CD113884 | Class5 | frameshift | 1 | PTV |
| *CHEK2* | NM_007194.3 | Chr22(GRCh37):g.29108007T>C | 6/15 | c.684-2 A>G | p.(?) | CS160107 | Class4 | splice defect | 1 | PTV |
| *CHEK2* | NM_007194.3 | Chr22(GRCh37):g.29099507A>C | 8/15 | c.894T>G | p.(Tyr298*) | / | Class5 | nonsense | 2 | PTV |
| *CHEK2* | NM_007194.3 | Chr22(GRCh37):g.29099499del | 8/15 | c.902del | p.(Leu301Trpfs*3) | CD159815 | Class5 | frameshift | 1 | PTV |
| *CHEK2* | NM_007194.3 | Chr22(GRCh37):g.29091857del | 11/15 | c.1100del | p.(Thr367Metfs*15) | CD993415 | Class5 | frameshift | 79 | PTV |
| *CHEK2* | NM_007194.3 | Chr22(GRCh37):g.29091193G>A | 12/15 | c.1297C>T | p.(Gln433*) | / | Class5 | nonsense | 1 | PTV |
| *CHEK2* | NM_007194.3 | Chr22(GRCh37):g.29091122dup | 12/15 | c.1368dup | p.(Glu457Argfs*33) | / | Class5 | frameshift | 1 | PTV |
| *CHEK2* | NM_007194.3 | Chr22(GRCh37):g.29090019C>T | intron13 | c.1461+1G>A | p.(?) | / | Class4 | splice defect | 1 | PTV |
| *NBN* | NM_002485.4 | Chr8(GRCh37):g.90994998del | 2/16 | c.123delC | p.(Ser42Alafs*7) | / | Class5 | frameshift | 1 | PTV |
| *NBN* | NM_002485.4 | Chr8(GRCh37):g.90993711_90993712insTC | 3/16 | c.211_212insGA | p.(Asn71Argfs*22) | / | Class5 | frameshift | 1 | PTV |
| *NBN* | NM_002485.4 | Chr8(GRCh37):g.90983442_90983446del | 6/16 | c.657_661delACAAA | p.(Lys219Asnfs*16) | CD982819 | Class5 | frameshift | 7 | PTV |
| *NBN* | NM_002485.4 | Chr8(GRCh37):g.90967766del | 10/16 | c.1141del | p.(Pro381Glnfs*23) | CD982822 | Class5 | frameshift | 1 | PTV |
| *NBN* | NM_002485.4 | Chr8(GRCh37):g.90967512del | 10/16 | c.1396del | p.(Arg466Glyfs*18) | / | Class5 | frameshift | 1 | PTV |
| *NBN* | NM_002485.4 | Chr8(GRCh37):g.90965666dup | 11/16 | c.1651dup | p.(Arg551Lysfs*5) | / | Class5 | frameshift | 1 | PTV |
| *PALB2* | NM_024675.3 | Chr16(GRCh37):g.23652458del | 1/13 | c.21del | p.(Lys7Asnfs*11) | / | Class5 | frameshift | 1 | PTV |
| *PALB2* | NM_024675.3 | Chr16(GRCh37):g.23649275T>C | intron2 | c.109-2A>G | p.(?) | / | Class4 | splice defect | 1 | PTV |
| *PALB2* | NM_024675.3 | Chr16(GRCh37):g.23649207_23649210del | 3/13 | c.172_175del | p.(Gln60Argfs*7) | CD092061 | Class5 | frameshift | 6 | PTV |
| *PALB2* | NM_024675.3 | Chr16(GRCh37):g.23649170C>A | intron3 | c.211+1G>T | p.(?) | / | Class4 | splice defect | 1 | PTV |
| *PALB2* | NM_024675.3 | Chr16(GRCh37):g.23647644T>A | 4/13 | c.223A>T | p.(Lys75*) | / | Class5 | nonsense | 1 | PTV |
| *PALB2* | NM_024675.3 | Chr16(GRCh37):g.23647357_23647358del | 4/13 | c.509_510del | p.(Arg170Ilefs*14) | CD100767 | Class5 | frameshift | 18 | PTV |
| *PALB2* | NM_024675.3 | Chr16(GRCh37):g.23647309del | 4/13 | c.558delT | p.(Pro187Leufs*6) | / | Class5 | frameshift | 1 | PTV |
| *PALB2* | NM_024675.3 | Chr16(GRCh37):g.23647266dup | 4/13 | c.601dup | p.(Ser201Lysfs*3) | / | Class5 | frameshift | 1 | PTV |
| *PALB2* | NM_024675.3 | Chr16(GRCh37):g.23647209del | 4/13 | c.658delA | p.(Ser220Valfs*3) | / | Class5 | frameshift | 1 | PTV |
| *PALB2* | NM_024675.3 | Chr16(GRCh37):g.23647109_23647110del | 4/13 | c.757_758del | p.(Leu253Ilefs*3) | CD070515 | Class5 | frameshift | 4 | PTV |
| *PALB2* | NM_024675.3 | Chr16(GRCh37):g.23647028del | 4/13 | c.839del | p.(Asn280Thrfs*8) | / | Class5 | frameshift | 1 | PTV |
| *PALB2* | NM_024675.3 | Chr16(GRCh37):g.23646927G>A | 4/13 | c.940C>T | p.(Gln314*) | / | Class5 | nonsense | 1 | PTV |
| *PALB2* | NM_024675.3 | Chr16(GRCh37):g.23646854del | 4/13 | c.1013delC | Pro338Glnfs*8) | / | Class4 | frameshift | 1 | PTV |
| *PALB2* | NM_024675.3 | Chr16(GRCh37):g.23646826_23646830del | 4/13 | c.1037_1041del | p.(Lys346Thrfs*13) | CD137939 | Class5 | frameshift | 1 | PTV |
| *PALB2* | NM_024675.3 | Chr16(GRCh37):g.23646828C>A | 4/13 | c.1039G>T | p.(Glu347*) | / | Class5 | nonsense | 1 | PTV |
| *PALB2* | NM_024675.3 | Chr16(GRCh37):g.23646821del | 4/13 | c.1046delA | p.(Asn349Ilefs*7) | CD1610912 | Class5 | frameshift | 1 | PTV |
| *PALB2* | NM_024675.3 | Chr16(GRCh37):g.23646627G>A | 4/13 | c.1240C>T | p.(Arg414*) | CM106645 | Class5 | nonsense | 1 | PTV |
| *PALB2* | NM_024675.3 | Chr16(GRCh37):g.23646443dup | 4/13 | c.1424dup | p.(Arg476Lysfs*11) | / | Class5 | frameshift | 1 | PTV |
| *PALB2* | NM_024675.3 | Chr16(GRCh37):g.23646316_23646317del | 4/13 | c.1550_1551delAA | p.(Lys517Ilefs*11) | / | Class5 | frameshift | 1 | PTV |
| *PALB2* | NM_024675.3 | Chr16(GRCh37):g.23641751C>T | 5/13 | c.1724G>A | p.(Trp575*) | / | Class5 | nonsense | 1 | PTV |
| *PALB2* | NM_024675.3 | Chr16(GRCh37):g.23641556G>T | 5/13 | c.1919C>A | p.(Ser640*) | CM158990 | Class5 | nonsense | 1 | PTV |
| *PALB2* | NM_024675.3 | Chr16(GRCh37):g.23641218G>A | 5/13 | c.2257C>T | p.(Arg753*) | CM070237 | Class5 | nonsense | 1 | PTV |
| *PALB2* | NM_024675.3 | Chr16(GRCh37):g.23641005dup | 5/13 | c.2470dup | p.(Cys824Leufs*2) | / | Class5 | frameshift | 1 | PTV |
| *PALB2* | NM_024675.3 | Chr16(GRCh37):g.23640959del | intron5 | c.2514+2del | p.(?) | / | Class4 | splice defect | 1 | PTV |
| *PALB2* | NM_024675.3 | Chr16(GRCh37):g.23637570C>T | 7/13 | c.2735G>A | p.(Trp912*) | / | Class5 | nonsense | 1 | PTV |
| *PALB2* | NM_024675.3 | Chr16(GRCh37):g.23637555A>T | intron7 | c.2748+2T>A | p.(?) | / | Class4 | splice defect | 1 | PTV |
| *PALB2* | NM_024675.3 | Chr16(GRCh37):g.23614979del | 13/13 | c.3361delG | p.(Gly1121Valfs*3) | CD137601 | Class5 | frameshift, affecting WD40 repeat- containing domain | 3 | PTV |
| *PALB2* | NM_024675.3 | Chr16(GRCh37):g.23614885dup | 13/13 | c.3456dup | p.(Pro1153Thrfs*4) | CI147535 | Class5 | frameshift, affecting WD40 repeat- containing domain | 6 | PTV |
|  |  |  |  |  |  |  |  |  |  |  |
| **gene** | **NCBI accession** | **genomic coordinates** | **exon/intron** | **HGVS (nucleotide)** | **HGVS (protein)** | **HGMD** | **classification** | **consequence** | **no. of patients** | **category** |
| *PALB2* | NM_024675.3 | Chr16(GRCh37):g.23614833_23614834del | 13/13 | c.3507_3508del | p.(His1170Phefs*19) | CD143102 | Class5 | frameshift, affecting WD40 repeat- containing domain | 1 | PTV |
| *PALB2* | NM_024675.3 | Chr16(GRCh37):g.23614792G>T | 13/13 | c.3549C>A | p.(Tyr1183*) | CM070239 | Class5 | nonsense, affecting WD40 repeat- containing domain | 4 | PTV |
| *RAD51C* | NM_058216.2 | Chr17(GRCh37):g.56772370dup | 2/9 | c.224dup | p.(Tyr75*) | CI102530 | Class5 | nonsense | 1 | PTV |
| *RAD51C* | NM_058216.2 | Chr17(GRCh37):g.56774174dup | 3/9 | c.525dup | p.(Cys176Leufs*27) | CI102531 | Class5 | frameshift | 1 | PTV |
| *RAD51C* | NM_058216.2 | Chr17(GRCh37):g.56787219G>A | intron4 | c.706-1G>A | p.(?) | / | Class4 | splice defect | 1 | PTV |
| *RAD51C* | NM_058216.2 | Chr17(GRCh37):g.56787218A>G | intron4 | c.706-2A>G | p.(?) | CS118878 | Class4 | splice defect | 3 | PTV |
| *RAD51C* | NM_058216.2 | Chr17(GRCh37):g.56798131del | 6/9 | c.862del | p.(Thr288Glnfs*14) | / | Class5 | frameshift | 1 | PTV |
| *RAD51C* | NM_058216.2 | Chr17(GRCh37):g.56770006_56801400del | intron6 | c.905-1_2del | p.(?) | CD159246 | Class4 | splice defect | 2 | PTV |
| *RAD51D* | NM_002878.3 | Chr17(GRCh37):g.33434127_33434130del | 5/10 | c.357_360del | p.(Cys119Trpfs*16) | CD1610024 | Class5 | frameshift | 1 | PTV |
| *RAD51D* | NM_002878.3 | Chr17(GRCh37):g.33430317G>A | 8/10 | c.694C>T | p.(Arg232*) | CM128411 | Class5 | nonsense | 4 | PTV |
| *TP53* | NM_000546.5 | Chr17(GRCh37):g.7577157T>C | intron7 | c.783-2A>G | p.(?) | CS169322 | Class4 | splice defect | 1 | PTV |
| *TP53* | NM_000546.5 | Chr17(GRCh37):g.7574003G>A | 10/11 | c.1024C>T | p.(Arg342*) | CM004908 | Class5 | nonsense | 2 | PTV |
| *ATM* | NM_000051.3 | Chr11(GRCh37):g.108188101C>A | 43/63 | c.6200C>A | p.(Ala2067Asp) | CM990216 | Class4 | missense | 2 | DV |
| *ATM* | NM_000051.3 | Chr11(GRCh37):g.108199839C>T | 49/63 | c.7181C>T | p.(Ser2394Leu) | CM083481 | Class4 | missense | 1 | DV |
| *ATM* | NM_000051.3 | Chr11(GRCh37):g.108203486T>G | intron52 | c.7789-3T>G | p.(?) | CS961481 | Class4 | splice defect | 2 | DV |
| *ATM* | NM_000051.3 | Chr11(GRCh37):g.108203575_108203576delinsGC | 53/63 | c.7875_7876delinsGC | p.(Asp2625_Ala2626delinsGluPro) | CX984027 | Class4 | missense | 3 | DV |
| *ATM* | NM_000051.3 | Chr11(GRCh37):g.108205832T>C | 55/63 | c.8147T>C | p.(Val2716Ala) | CM025322 | Class4 | missense | 2 | DV |
| *CDH1* | NM_004360.3 | Chr16(GRCh37):g.68853296C>G | 11/16 | c.1679C>G | p.(Thr560Arg) | CM135593 | Class4 | missense | 1 | DV |
| *CHEK2* | NM_007194.3 | Chr22(GRCh37):g.29121326T>C | 3/15 | c.349A>G | p.(Arg117Gly) | CM023898 | Class4 | missense | 8 | DV |
| *CHEK2* | NM_007194.3 | Chr22(GRCh37):g.29121242G>A | 3/15 | c.433C>T | p.(Arg145Trp) | CM013593 | Class4 | missense | 3 | DV |
| *CHEK2* | NM_007194.3 | Chr22(GRCh37):g.29121058C>T | 4/15 | c.499G>A | p.(Gly167Arg) | CM030416 | Class4 | missense | 3 | DV |
| *CHEK2* | NM_007194.3 | Chr22(GRCh37):g.29120962T>A | intron4 | c.592+3A>T | p.(?) | CS1610907 | Class4 | splice defect | 3 | DV |
| *CHEK2* | NM_007194.3 | Chr22(GRCh37):g.29091788T>G | 11/15 | c.1169A>C | p.(Tyr390Ser) | CM119701 | Class4 | missense | 4 | DV |
| *CHEK2* | NM_007194.3 | Chr22(GRCh37):g.29091220A>G | 12/15 | c.1270T>C | p.(Tyr424His) | CM124200 | Class4 | missense | 1 | DV |
| *CHEK2* | NM_007194.3 | Chr22(GRCh37):g.29090054G>A | 13/15 | c.1427C>T | p.(Thr476Met) | CM119709 | Class4 | missense | 13 | DV |
| *PALB2* | NM_024675.3 | Chr16(GRCh37):g.23652461C>A | 1/13 | c.18G>T | p.(=) | / | Class4 | splice defect | 2 | DV |
| *PALB2* | NM_024675.3 | Chr16(GRCh37):g.23632678C>G | intron10 | c.3113+5G>C | p.(?) | CS070398 | Class4 | splice defect | 1 | DV |
| *RAD51C* | NM_058216.2 | Chr17(GRCh37):g.56774077A>G | 3/9 | c.428A>G | p.(Gln143Arg) | CM123221 | Class5 | missense | 2 | DV |
| *RAD51D* | NM_002878.3 | Chr17(GRCh37):g.33428327G>A | 9/10 | c.796C>T | p.(Arg266Cys) | CM130556 | Class4 | missense | 1 | DV |
| *TP53* | NM_000546.5 | Chr17(GRCh37):g.7578413C>T | 5/11 | c.517G>A | p.(Val173Met) | CM070299 | Class5 | missense | 1 | DV |
| *TP53* | NM_000546.5 | Chr17(GRCh37):g.7578395G>A | 5/11 | c.535C>T | p.(His179Tyr) | CM067054 | Class4 | missense | 1 | DV |
| *TP53* | NM_000546.5 | Chr17(GRCh37):g.7578388C>T | 5/11 | c.542G>A | p.(Arg181His) | CM920671 | Class4 | missense | 1 | DV |
| *TP53* | NM_000546.5 | Chr17(GRCh37):g.7577545T>A | 7/11 | c.736A>T | p.(Met246Leu) | / | Class4 | missense | 1 | DV |
| *TP53* | NM_000546.5 | Chr17(GRCh37):g.7577539G>A | 7/11 | c.742C>T | p.(Arg248Trp) | CM900211 | Class4 | missense | 3 | DV |
| *TP53* | NM_000546.5 | Chr17(GRCh37):g.7577139G>A | 8/11 | c.799C>T | p.(Arg267Trp) | CM120851 | Class4 | missense | 1 | DV |
| *TP53* | NM_000546.5 | Chr17(GRCh37):g.7577120C>T | 8/11 | c.818G>A | p.(Arg273His) | CM920677 | Class5 | missense | 1 | DV |
| *TP53* | NM_000546.5 | Chr17(GRCh37):g.7577106G>A | 8/11 | c.832C>T | p.(Pro278Ser) | CM011015 | Class4 | missense | 1 | DV |
| *TP53* | NM_000546.5 | Chr17(GRCh37):g.7577094G>A | 8/11 | c.844C>T | p.(Arg282Trp) | CM920678 | Class4 | missense | 1 | DV |
| *TP53* | NM_000546.5 | Chr17(GRCh37):g.7574018G>A | 10/11 | c.1009C>T | p.(Arg337Cys) | CM981929 | Class4 | missense | 1 | DV |
| *TP53* | NM_000546.5 | Chr17(GRCh37):g.7573987G>T | 10/11 | c.1040C>A | p.(Ala347Asp) | CM169324 | Class5 | missense | 2 | DV |
| *ATM* | NM_000051.3 | Chr11(GRCh37):g.108098540C>T | 3/63 | c.110C>T | p.(Pro37Leu) | / | Class3 | missense | 1 | VUS |
| *ATM* | NM_000051.3 | Chr11(GRCh37):g.108098555A>G | 3/63 | c.125A>G | p.(His42Arg) | / | Class3 | missense | 1 | VUS |
| *ATM* | NM_000051.3 | Chr11(GRCh37):g.108098563C>T | 3/63 | c.133C>T | p.(Arg45Trp) | / | Class3 | missense | 1 | VUS |
| *ATM* | NM_000051.3 | Chr11(GRCh37):g.108099919A>G | 4/63 | c.200A>G | p.(Tyr67Cys) | / | Class3 | missense | 2 | VUS |
| *ATM* | NM_000051.3 | Chr11(GRCh37):g.108099999A>G | 4/63 | c.280A>G | p.(Met94Val) | / | Class3 | missense | 1 | VUS |
| *ATM* | NM_000051.3 | Chr11(GRCh37):g.108106441G>C | 4/63 | c.295A>G | p.(Ser99Gly) | / | Class3 | missense | 1 | VUS |
| *ATM* | NM_000051.3 | Chr11(GRCh37):g.108114722A>G | 4/63 | c.322G>A | p.(Ala108Thr) | / | Class3 | missense | 1 | VUS |
| *ATM* | NM_000051.3 | Chr11(GRCh37):g.108114725A>T | 5/63 | c.376G>C | p.(Asp126His) | / | Class3 | missense | 1 | VUS |
| *ATM* | NM_000051.3 | Chr11(GRCh37):g.108117798C>T | 6/63 | c.508G>T | p.(Val170Leu) | / | Class3 | missense | 1 | VUS |
| *ATM* | NM_000051.3 | Chr11(GRCh37):g.108117799G>A | 6/63 | c.539A>G | p.(Gln180Arg) | / | Class3 | missense | 1 | VUS |
| *ATM* | NM_000051.3 | Chr11(GRCh37):g.108121564T>C | 6/63 | c.542A>T | p.(Asp181Val) | / | Class3 | missense | 1 | VUS |
| **gene** | **NCBI accession** | **genomic coordinates** | **exon/intron** | **HGVS (nucleotide)** | **HGVS (protein)** | **HGMD** | **classification** | **consequence** | **no. of patients** | **category** |
| *ATM* | NM_000051.3 | Chr11(GRCh37):g.108121636A>C | 6/63 | c.610G>A | p.(Gly204Arg) | / | Class3 | missense | 1 | VUS |
| *ATM* | NM_000051.3 | Chr11(GRCh37):g.108122659G>A | 6/63 | c.620C>G | p.(Ser207Cys) | / | Class3 | missense | 1 | VUS |
| *ATM* | NM_000051.3 | Chr11(GRCh37):g.108122704A<G | 7/63 | c.670A>G | p.(Lys224Glu) | CM000650 | Class3 | missense | 4 | VUS |
| *ATM* | NM_000051.3 | Chr11(GRCh37):g.108122704A>G | 7/63 | c.737A>G | p.(Asn246Ser) | / | Class3 | missense | 1 | VUS |
| *ATM* | NM_000051.3 | Chr11(GRCh37):g.108123551C>T | 8/63 | c.902G>A | p.(Gly301Asp) | CM0910482 | Class3 | missense | 1 | VUS |
| *ATM* | NM_000051.3 | Chr11(GRCh37):g.108128246T>A | 8/63 | c.998C>T | p.(Ser333Phe) | CM152767 | Class3 | missense | 12 | VUS |
| *ATM* | NM_000051.3 | Chr11(GRCh37):g.108128290A>G | 8/63 | c.1009C>T | p.(Arg337Cys) | CM0910483 | Class3 | missense | 2 | VUS |
| *ATM* | NM_000051.3 | Chr11(GRCh37):g.108137926G>C | 8/63 | c.1010G>A | p.(Arg337His) | / | Class3 | missense | 2 | VUS |
| *ATM* | NM_000051.3 | Chr11(GRCh37):g.108137962G>A | intron8 | c.1066-6T>G | p.(?) | CS000846 | Class3 | splice defect? | 23 | VUS |
| *ATM* | NM_000051.3 | Chr11(GRCh37):g.108137970A>T | 9/63 | c.1078G>C | p.(Asp360His) | / | Class3 | missense | 1 | VUS |
| *ATM* | NM_000051.3 | Chr11(GRCh37):g.108139160G>C | 9/63 | c.1194T>A | p.(Asp389Glu) | / | Class3 | missense | 1 | VUS |
| *ATM* | NM_000051.3 | Chr11(GRCh37):g.108139226A>G | 10/63 | c.1372T>C | p.(Cys458Arg) | / | Class3 | missense | 1 | VUS |
| *ATM* | NM_000051.3 | Chr11(GRCh37):g.108139319T>G | 10/63 | c.1379C>T | p.(Thr460Met) | / | Class3 | missense | 1 | VUS |
| *ATM* | NM_000051.3 | Chr11(GRCh37):g.108141988T>C | 10/63 | c.1444A>C | p.(Lys482Gln) | / | Class3 | missense | 2 | VUS |
| *ATM* | NM_000051.3 | Chr11(GRCh37):g.108142070A>G | 10/63 | c.1516G>T | p.(Gly506Cys) | / | Class3 | missense | 1 | VUS |
| *ATM* | NM_000051.3 | Chr11(GRCh37):g.108143259G>T | 10/63 | c.1543A>G | p.(Ser515Gly) | / | Class3 | missense | 1 | VUS |
| *ATM* | NM_000051.3 | Chr11(GRCh37):g.108151756A>G | 10/63 | c.1595G>A | p.(Cys532Tyr) | / | Class3 | missense | 4 | VUS |
| *ATM* | NM_000051.3 | Chr11(GRCh37):g.108151884C>T | 11/63 | c.1703G>A | p.(Arg568Lys) | / | Class3 | missense | 4 | VUS |
| *ATM* | NM_000051.3 | Chr11(GRCh37):g.108155132G>A | 11/63 | c.1748A>G | p.(Tyr583Cys) | / | Class3 | missense | 3 | VUS |
| *ATM* | NM_000051.3 | Chr11(GRCh37):g.108160329G>A | 12/63 | c.1810C>T | p.(Pro604Ser) | CM045888 | Class3 | missense | 13 | VUS |
| *ATM* | NM_000051.3 | Chr11(GRCh37):g.108160416T>C | 13/63 | c.1943T>C | p.(Val648Ala) | / | Class3 | missense | 1 | VUS |
| *ATM* | NM_000051.3 | Chr11(GRCh37):g.108160454A>C | 13/63 | c.1996G>C | p.(Val666Leu) | / | Class3 | missense | 1 | VUS |
| *ATM* | NM_000051.3 | Chr11(GRCh37):g.108160516A>G | 14/63 | c.2127T>G | p.(lle709Met) | / | Class3 | missense | 1 | VUS |
| *ATM* | NM_000051.3 | Chr11(GRCh37):g.108163414G>T | 14/63 | c.2158C>T | p.(Arg720Cys) | CM0910489 | Class3 | missense | 1 | VUS |
| *ATM* | NM_000051.3 | Chr11(GRCh37):g.108163486C>T | 15/63 | c.2289T>A | p.(Phe763Leu) | / | Class3 | missense | 5 | VUS |
| *ATM* | NM_000051.3 | Chr11(GRCh37):g.108164059A>G | 15/63 | c.2333A>G | p.(Asn778Ser) | / | Class3 | missense | 1 | VUS |
| *ATM* | NM_000051.3 | Chr11(GRCh37):g.108164137T>C | intron15 | c.2377-6T>A | p.(?) | / | Class3 | splice defect? | 1 | VUS |
| *ATM* | NM_000051.3 | Chr11(GRCh37):g.108164196C>T | 16/63 | c.2423C>T | p.(Thr808Ile) | / | Class3 | missense | 1 | VUS |
| *ATM* | NM_000051.3 | Chr11(GRCh37):g.108172434G>T | 16/63 | c.2428A>G | p.(Lys810Glu) | / | Class3 | missense | 1 | VUS |
| *ATM* | NM_000051.3 | Chr11(GRCh37):g.108173736T>G | 17/63 | c.2495G>C | p.(Arg832Pro) | / | Class3 | missense | 1 | VUS |
| *ATM* | NM_000051.3 | Chr11(GRCh37):g.108180945G>C | 17/63 | c.2519A>T | p.(Asp840Val) | / | Class3 | missense | 2 | VUS |
| *ATM* | NM_000051.3 | Chr11(GRCh37):g.108183157G>A | 17/63 | c.2531G>A | p.(Gly844Glu) | / | Class3 | missense | 2 | VUS |
| *ATM* | NM_000051.3 | Chr11(GRCh37):g.108196162T>C | 17/63 | c.2539A>T | p.(Met847Leu) | / | Class3 | missense | 1 | VUS |
| *ATM* | NM_000051.3 | Chr11(GRCh37):g.108196837G>A | 18/63 | c.2662G>C | p.(Glu888Gln) | / | Class3 | missense | 1 | VUS |
| *ATM* | NM_000051.3 | Chr11(GRCh37):g.108196837G>C | 18/63 | c.2728A>G | p.(Thr910Ala) | / | Class3 | missense | 1 | VUS |
| *ATM* | NM_000051.3 | Chr11(GRCh37):g.108196935G>T | 18/63 | c.2770C>T | p.(Arg924Trp) | / | Class3 | missense | 5 | VUS |
| *ATM* | NM_000051.3 | Chr11(GRCh37):g.108198394C>A | 18/63 | c.2821T>G | p.(Ser941Ala) | / | Class3 | missense | 1 | VUS |
| *ATM* | NM_000051.3 | Chr11(GRCh37):g.108198396T>C | 18/63 | c.2836A>G | p.(Met946Val) | / | Class3 | missense | 1 | VUS |
| *ATM* | NM_000051.3 | Chr11(GRCh37):g.108199845C>G | 20/63 | c.2932T>C | p.(Ser978Pro) | CM1410494 | Class3 | missense | 9 | VUS |
| *ATM* | NM_000051.3 | Chr11(GRCh37):g.108199938T>G | 20/63 | c.3014A>G | p.(Asn1005Ser) | / | Class3 | missense | 1 | VUS |
| *ATM* | NM_000051.3 | Chr11(GRCh37):g.108200961G>A | 21/63 | c.3078G>T | p.(Trp1026Cys) | / | Class3 | missense | 1 | VUS |
| *ATM* | NM_000051.3 | Chr11(GRCh37):g.108200997T>A | 21/63 | c.3118A>G | p.(Met1040Val) | / | Class3 | missense | 1 | VUS |
| *ATM* | NM_000051.3 | Chr11(GRCh37):g.108201023T>C | 22/63 | c.3176C>T | p.(Ala1059Val) | / | Class3 | missense | 1 | VUS |
| *ATM* | NM_000051.3 | Chr11(GRCh37):g.108201032G>A | 23/63 | c.3378A>G | p.(Lys1126Lys) | / | Class3 | missense | 1 | VUS |
| *ATM* | NM_000051.3 | Chr11(GRCh37):g.108201108T>G | intron23 | c.3403-3A>C | p.(?) | / | Class3 | splice defect? | 1 | VUS |
| *ATM* | NM_000051.3 | Chr11(GRCh37):g.108202273G>A | 24/63 | c.3437A>G | p.(Glu1146Gly) | / | Class3 | missense | 1 | VUS |
| *ATM* | NM_000051.3 | Chr11(GRCh37):g.108202277T>G | 24/63 | c.3457G>C | p.(Val1153Leu) | / | Class3 | missense | 1 | VUS |
| *ATM* | NM_000051.3 | Chr11(GRCh37):g.108203518A>G | 24/63 | c.3478G>C | p.(Val1160Leu) | / | Class3 | missense | 1 | VUS |
| *ATM* | NM_000051.3 | Chr11(GRCh37):g.108205756C>T | 24/63 | c.3565C>T | p.(Leu1189Phe) | / | Class3 | missense | 1 | VUS |
| *ATM* | NM_000051.3 | Chr11(GRCh37):g.108205835A>G | 25/63 | c.3630G>A | p.(Met1210Ile) | CM023333 | Class3 | missense | 1 | VUS |
| *ATM* | NM_000051.3 | Chr11(GRCh37):g.108206634A>C | 25/63 | c.3689A>G | p.(Asn1230Ser) | / | Class3 | missense | 1 | VUS |
| *ATM* | NM_000051.3 | Chr11(GRCh37):g.108224555A>G | 25/63 | c.3709A>T | p.(Ile1237Phe) | / | Class3 | missense | 1 | VUS |
| *ATM* | NM_000051.3 | Chr11(GRCh37):g.108235832T>A | 25/63 | c.3729T>A | p.(Asn1243Lys) | / | Class3 | missense | 3 | VUS |
| *ATM* | NM_000051.3 | Chr11(GRCh37):g.108154962A>G | 26/63 | c.3755A>G | p.(Tyr1252Cys) | / | Class3 | missense | 1 | VUS |
| **gene** | **NCBI accession** | **genomic coordinates** | **exon/intron** | **HGVS (nucleotide)** | **HGVS (protein)** | **HGMD** | **classification** | **consequence** | **no. of patients** | **category** |
| *ATM* | NM_000051.3 | Chr11(GRCh37):g.108155009G>A | 26/63 | c.3802G>A | p.(Val1268Met) | / | Class3 | missense | 1 | VUS |
| *ATM* | NM_000051.3 | Chr11(GRCh37):g.108155033C>G | 26/63 | c.3826C>G | p.(Gln1276Glu) | / | Class3 | missense | 1 | VUS |
| *ATM* | NM_000051.3 | Chr11(GRCh37):g.108155039G>A | 26/63 | c.3832G>A | p.(Asp1278Asn) | / | Class3 | missense | 1 | VUS |
| *ATM* | NM_000051.3 | Chr11(GRCh37):g.108155049G>A | 26/63 | c.3842G>A | p.(Ser1281Asn) | / | Class3 | missense | 1 | VUS |
| *ATM* | NM_000051.3 | Chr11(GRCh37):g.108155132G>A | 26/63 | c.3925G>A | p.(Ala1309Thr) | / | Class3 | missense | 13 | VUS |
| *ATM* | NM_000051.3 | Chr11(GRCh37):g.108155199A>G | 26/63 | c.3992A>G | p.(Gln1331Arg) | / | Class3 | missense | 1 | VUS |
| *ATM* | NM_000051.3 | Chr11(GRCh37):g.108158393C>A | 27/63 | c.4060C>A | p.(Pro1354Thr) | / | Class3 | missense | 1 | VUS |
| *ATM* | NM_000051.3 | Chr11(GRCh37):g.108160329G>A | 29/63 | c.4237G>A | p.(Asp1413Asn) | / | Class3 | missense | 1 | VUS |
| *ATM* | NM_000051.3 | Chr11(GRCh37):g.108160416T>C | 29/63 | c.4324T>C | p.(Tyr1442His) | CM0910502 | Class3 | missense | 7 | VUS |
| *ATM* | NM_000051.3 | Chr11(GRCh37):g.108160454A>C | 29/63 | c.4362A>C | p.(Lys1454Asn) | CM0910503 | Class3 | missense | 3 | VUS |
| *ATM* | NM_000051.3 | Chr11(GRCh37):g.108160480T>G | 29/63 | c.4388T>G | p.(Phe1463Cys) | CM022199 | Class3 | missense | 4 | VUS |
| *ATM* | NM_000051.3 | Chr11(GRCh37):g.108160516A>G | 29/63 | c.4424A>G | p.(Tyr1475Cys) | / | Class3 | missense | 12 | VUS |
| *ATM* | NM_000051.3 | Chr11(GRCh37):g.108163414G>T | 30/63 | c.4505G>T | p.(Cys1502Phe) | / | Class3 | missense | 2 | VUS |
| *ATM* | NM_000051.3 | Chr11(GRCh37):g.108163486C>T | 30/63 | c.4577C>T | p.(Pro1526Leu) | / | Class3 | missense | 1 | VUS |
| *ATM* | NM_000051.3 | Chr11(GRCh37):g.108164059A>G | 31/63 | c.4631A>G | p.(Tyr1544Cys) | / | Class3 | missense | 2 | VUS |
| *ATM* | NM_000051.3 | Chr11(GRCh37):g.108164137T>C | 31/63 | c.4709T>C | p.(Val1570Ala) | CM016180 | Class3 | missense | 10 | VUS |
| *ATM* | NM_000051.3 | Chr11(GRCh37):g.108164152G>A | 31/63 | c.4724G>A | p.(Arg1575His) | CM0910508 | Class3 | missense | 1 | VUS |
| *ATM* | NM_000051.3 | Chr11(GRCh37):g.108164196C>T | 31/63 | c.4768C>T | p.(Leu1590Phe) | / | Class3 | missense | 5 | VUS |
| *ATM* | NM_000051.3 | Chr11(GRCh37):g.108165750A>G | 32/63 | c.4873A>G | p.(Lys1625Glu) | / | Class3 | missense | 1 | VUS |
| *ATM* | NM_000051.3 | Chr11(GRCh37):g.108168079A>G | 33/63 | c.4975A>G | p.(Ile1659Val) | / | Class3 | missense | 1 | VUS |
| *ATM* | NM_000051.3 | Chr11(GRCh37):g.108172382G>C | 35/63 | c.5185G>C | p.(Val1729Leu) | / | Class3 | missense | 1 | VUS |
| *ATM* | NM_000051.3 | Chr11(GRCh37):g.108172434G>T | 35/63 | c.5237G>T | p.(Gly1746Val) | / | Class3 | missense | 1 | VUS |
| *ATM* | NM_000051.3 | Chr11(GRCh37):g.108172459G>T | 35/63 | c.5262G>T | p.(Lys1754Asn) | CM035779 | Class3 | missense | 1 | VUS |
| *ATM* | NM_000051.3 | Chr11(GRCh37):g.108172460A>G | 35/63 | c.5263A>G | p.(Met1755Val) | / | Class3 | missense | 1 | VUS |
| *ATM* | NM_000051.3 | Chr11(GRCh37):g.108173736T>G | 36/63 | c.5476T>G | p.(Leu1826Val) | / | Class3 | missense | 1 | VUS |
| *ATM* | NM_000051.3 | Chr11(GRCh37):g.108175490T>A | 37/63 | c.5585T>A | p.(Leu1862His) | / | Class3 | missense | 1 | VUS |
| *ATM* | NM_000051.3 | Chr11(GRCh37):g.108175523G>A | 37/63 | c.5618G>A | p.(Cys1873Tyr) | / | Class3 | missense | 1 | VUS |
| *ATM* | NM_000051.3 | Chr11(GRCh37):g.108178699G>A | 38/63 | c.5750G>A | p.(Arg1917Lys) | / | Class3 | missense | 1 | VUS |
| *ATM* | NM_000051.3 | Chr11(GRCh37):g.108180945G>C | 39/63 | c.5821G>C | p.(Val1941Leu) | CM094667 | Class3 | missense | 1 | VUS |
| *ATM* | NM_000051.3 | Chr11(GRCh37):g.108181014A>G | 39/63 | c.5890A>G | p.(Lys1964Glu) | CM0910514 | Class3 | missense | 2 | VUS |
| *ATM* | NM_000051.3 | Chr11(GRCh37):g.108181027A>G | 39/63 | c.5903A>G | p.(Asp1968Gly) | / | Class3 | missense | 1 | VUS |
| *ATM* | NM_000051.3 | Chr11(GRCh37):g.108183157G>A | 40/63 | c.5938G>A | p.(Gly1980Arg) | / | Class3 | missense | 1 | VUS |
| *ATM* | NM_000051.3 | Chr11(GRCh37):g.108183194A>C | 40/63 | c.5975A>C | p.(Lys1992Thr) | / | Class3 | missense | 2 | VUS |
| *ATM* | NM_000051.3 | Chr11(GRCh37):g.108186642A>G | intron41 | c.6095+4A>G | p.(?) | / | Class3 | splice defect? | 1 | VUS |
| *ATM* | NM_000051.3 | Chr11(GRCh37):g.108188116G>C | 43/63 | c.6215G>C | p.(Gly2072Ala) | / | Class3 | missense | 1 | VUS |
| *ATM* | NM_000051.3 | Chr11(GRCh37):g.108188158A>T | 43/63 | c.6257A>T | p.(Tyr2086Phe) | / | Class3 | missense | 1 | VUS |
| *ATM* | NM_000051.3 | Chr11(GRCh37):g.108192137C>A | 45/63 | c.6562C>A | p.(Leu2188Ile) | / | Class3 | missense | 1 | VUS |
| *ATM* | NM_000051.3 | Chr11(GRCh37):g.108196068T>G | 46/63 | c.6604T>G | p.(Tyr2202Asp) | / | Class3 | missense | 1 | VUS |
| *ATM* | NM_000051.3 | Chr11(GRCh37):g.108196162T>C | 46/63 | c.6698T>C | p.(Ile2233Thr) | / | Class3 | missense | 1 | VUS |
| *ATM* | NM_000051.3 | Chr11(GRCh37):g.108196797G>A | 47/63 | c.6820G>A | p.(Ala2274Thr) | CM016181 | Class3 | missense | 1 | VUS |
| *ATM* | NM_000051.3 | Chr11(GRCh37):g.108196837G>A | 47/63 | c.6860G>A | p.(Gly2287Glu) | / | Class3 | missense | 1 | VUS |
| *ATM* | NM_000051.3 | Chr11(GRCh37):g.108196837G>C | 47/63 | c.6860G>C | p.(Gly2287Ala) | / | Class3 | missense | 6 | VUS |
| *ATM* | NM_000051.3 | Chr11(GRCh37):g.108196896C>T | 47/63 | c.6919C>T | p.(Leu2307Phe) | CM152448 | Class3 | missense | 1 | VUS |
| *ATM* | NM_000051.3 | Chr11(GRCh37):g.108196935G>T | 47/63 | c.6958G>T | p.(Asp2320Tyr) | / | Class3 | missense | 1 | VUS |
| *ATM* | NM_000051.3 | Chr11(GRCh37):g.108198379C>T | 47/63 | c.6983C>T | p.(Pro2328Leu) | / | Class3 | missense | 1 | VUS |
| *ATM* | NM_000051.3 | Chr11(GRCh37):g.108198394C>A | 48/63 | c.6998C>A | p.(Thr2333Lys) | / | Class3 | missense | 1 | VUS |
| *ATM* | NM_000051.3 | Chr11(GRCh37):g.108198396T>C | 48/63 | c.7000T>C | p.(Tyr2334His) | / | Class3 | missense | 1 | VUS |
| *ATM* | NM_000051.3 | Chr11(GRCh37):g.108199845C>G | 49/63 | c.7187C>G | p.(Thr2396Ser) | CM092586 | Class3 | missense | 1 | VUS |
| *ATM* | NM_000051.3 | Chr11(GRCh37):g.108199893A>G | 49/63 | c.7235A>G | p.(Asn2412Ser) | / | Class3 | missense | 1 | VUS |
| *ATM* | NM_000051.3 | Chr11(GRCh37):g.108199923A>C | 49/63 | c.7265A>C | p.(Glu2422Ala) | / | Class3 | missense | 1 | VUS |
| *ATM* | NM_000051.3 | Chr11(GRCh37):g.108199938T>G | 49/63 | c.7280T>G | p.(Leu2427Arg) | / | Class3 | missense | 1 | VUS |
| *ATM* | NM_000051.3 | Chr11(GRCh37):g.108200961G>A | 50/63 | c.7328G>A | p.(Arg2443Gln) | / | Class3 | missense | 1 | VUS |
| *ATM* | NM_000051.3 | Chr11(GRCh37):g.108200997T>A | 50/63 | c.7364T>A | p.(Leu2455Gln) | / | Class3 | missense | 1 | VUS |
| *ATM* | NM_000051.3 | Chr11(GRCh37):g.108201008C>T | 50/63 | c.7375C>T | p.(Arg2459Cys) | / | Class3 | missense | 1 | VUS |
| **gene** | **NCBI accession** | **genomic coordinates** | **exon/intron** | **HGVS (nucleotide)** | **HGVS (protein)** | **HGMD** | **classification** | **consequence** | **no. of patients** | **category** |
| *ATM* | NM_000051.3 | Chr11(GRCh37):g.108201023T>C | 50/63 | c.7390T>C | p.(Cys2464Arg) | CM016183 | Class3 | missense | 17 | VUS |
| *ATM* | NM_000051.3 | Chr11(GRCh37):g.108201032G>A | 50/63 | c.7399G>A | p.(Val2467Ile) | / | Class3 | missense | 1 | VUS |
| *ATM* | NM_000051.3 | Chr11(GRCh37):g.108201096G>A | 50/63 | c.7463G>A | p.(Cys2488Tyr) | CM1314563 | Class3 | missense | 2 | VUS |
| *ATM* | NM_000051.3 | Chr11(GRCh37):g.108201108T>G | 50/63 | c.7475T>G | p.(Leu2492Arg) | / | Class3 | missense | 6 | VUS |
| *ATM* | NM_000051.3 | Chr11(GRCh37):g.108202223T>G | 51/63 | c.7568T>G | p.(Leu2523Trp) | / | Class3 | missense | 1 | VUS |
| *ATM* | NM_000051.3 | Chr11(GRCh37):g.108202226C>T | 51/63 | c.7571C>T | p.(Ala2524Val) | / | Class3 | missense | 1 | VUS |
| *ATM* | NM_000051.3 | Chr11(GRCh37):g.108202247T>C | 51/63 | c.7592T>C | p.(Met2531Thr) | CM0910522 | Class3 | missense | 1 | VUS |
| *ATM* | NM_000051.3 | Chr11(GRCh37):g.108202261G>A | 51/63 | c.7606G>A | p.(Gly2536Arg) | / | Class3 | missense | 1 | VUS |
| *ATM* | NM_000051.3 | Chr11(GRCh37):g.108202273G>A | 51/63 | c.7618G>A | p.(Val2540Ile) | / | Class3 | missense | 1 | VUS |
| *ATM* | NM_000051.3 | Chr11(GRCh37):g.108202277T>G | 51/63 | c.7622T>G | p.(Leu2541Arg) | CM095586 | Class3 | missense | 1 | VUS |
| *ATM* | NM_000051.3 | Chr11(GRCh37):g.108202667C>T | 52/63 | c.7691C>T | p.(Ala2564Val) | / | Class3 | missense | 1 | VUS |
| *ATM* | NM_000051.3 | Chr11(GRCh37):g.108203518A>G | 53/63 | c.7818A>G | p.(Ile2606Met) | / | Class3 | missense | 1 | VUS |
| *ATM* | NM_000051.3 | Chr11(GRCh37):g.108203565C>G | 53/63 | c.7865C>G | p.(Ala2622Gly) | / | Class3 | missense | 1 | VUS |
| *ATM* | NM_000051.3 | Chr11(GRCh37):g.108203619C>T | 53/63 | c.7919C>T | p.(Thr2640Ile) | / | Class3 | missense | 4 | VUS |
| *ATM* | NM_000051.3 | Chr11(GRCh37):g.108204663G>A | 54/63 | c.7978G>A | p.(Glu2660Lys) | / | Class3 | missense | 3 | VUS |
| *ATM* | NM_000051.3 | Chr11(GRCh37):g.108205700A>C | 55/63 | c.8015A>C | p.(Asp2672Ala) | / | Class3 | missense | 1 | VUS |
| *ATM* | NM_000051.3 | Chr11(GRCh37):g.108205756C>T | 55/63 | c.8071C>T | p.(Arg2691Cys) | CM035637 | Class3 | missense | 1 | VUS |
| *ATM* | NM_000051.3 | Chr11(GRCh37):g.108205835A>G | 55/63 | c.8150A>G | p.(Lys2717Arg) | CS961483 | Class3 | missense | 1 | VUS |
| *ATM* | NM_000051.3 | Chr11(GRCh37):g.108206634A>C | 56/63 | c.8214A>C | p.(Leu2738Phe) | / | Class3 | missense | 1 | VUS |
| *ATM* | NM_000051.3 | Chr11(GRCh37):g.108206648C>T | 56/63 | c.8228C>T | p.(Thr2743Met) | / | Class3 | missense | 1 | VUS |
| *ATM* | NM_000051.3 | Chr11(GRCh37):g.108213994G>A | 57/63 | c.8314G>A | p.(Gly2772Arg) | CM016184 | Class3 | missense | 1 | VUS |
| *ATM* | NM_000051.3 | Chr11(GRCh37):g.108216479A>C | 58/63 | c.8428A>C | p.(Lys2810Gln) | / | Class3 | missense | 2 | VUS |
| *ATM* | NM_000051.3 | Chr11(GRCh37):g.108216611C>T | 58/63 | c.8560C>T | p.(Arg2854Cys) | CM092588 | Class3 | missense | 1 | VUS |
| *ATM* | NM_000051.3 | Chr11(GRCh37):g.108216632A>G | 58/63 | c.8581A>G | p.(Ile2861Val) | CM1613045 | Class3 | missense | 1 | VUS |
| *ATM* | NM_000051.3 | Chr11(GRCh37):g.108218045A>C | 59/63 | c.​8624A>C | p.(​Asn2875Thr) | / | Class3 | missense | 1 | VUS |
| *ATM* | NM_000051.3 | Chr11(GRCh37):g.108218056A>G | 59/63 | c.8635A>G | p.(Asn2879Asp) | / | Class3 | missense | 1 | VUS |
| *ATM* | NM_000051.3 | Chr11(GRCh37):g.108218101T>G | intron59 | c.8671+9T>G | p.(?) | / | Class3 | splice defect? | 1 | VUS |
| *ATM* | NM_000051.3 | Chr11(GRCh37):g.108224555A>G | 60/63 | c.8734A>G | p.(Arg2912Gly) | CM014034 | Class3 | missense | 4 | VUS |
| *ATM* | NM_000051.3 | Chr11(GRCh37):g.108225557G>C | 61/63 | c.8806G>C | p.(Glu2936Gln) | / | Class3 | missense | 1 | VUS |
| *ATM* | NM_000051.3 | Chr11(GRCh37):g.108235832T>A | 62/63 | c.8874T>A | p.(Phe2958Leu) | / | Class3 | missense | 1 | VUS |
| *ATM* | NM_000051.3 | Chr11(GRCh37):g.108235853G>C | 62/63 | c.8895G>C | p.(Leu2965Phe) | / | Class3 | missense | 2 | VUS |
| *ATM* | NM_000051.3 | Chr11(GRCh37):g.108235879C>T | 62/63 | c.8921C>T | p.(Pro2974Leu) | / | Class3 | missense | 1 | VUS |
| *ATM* | NM_000051.3 | Chr11(GRCh37):g.108235896C>A | 62/63 | c.8938C>A | p.(Leu2980Ile) | / | Class3 | missense | 1 | VUS |
| *ATM* | NM_000051.3 | Chr11(GRCh37):g.108236150G>A | 63/63 | c.9086G>A | p.(Gly3029Asp) | / | Class3 | missense | 1 | VUS |
| *ATM* | NM_000051.3 | Chr11(GRCh37):g.108236173C>T | 63/63 | c.9109C>T | p.(Gln3037*) | / | Class3 | nonsense, 60nt/20aa upstream of the stop codon | 1 | VUS |
| *ATM* | NM_000051.3 | Chr11(GRCh37):g.108236230G>T | 63/63 | c.9166G>T | p.(Val3056Leu) | / | Class3 | missense | 1 | VUS |
| *CDH1* | NM_004360.3 | Chr16(GRCh37):g.68771326C>G | 1/16 | c.8C>G | p.(Pro3Arg) | CM110009 | Class3 | missense | 7 | VUS |
| *CDH1* | NM_004360.3 | Chr16(GRCh37):g.68772281C>A | 2/16 | c.130C>A | p.(Arg44Ser) | / | Class3 | missense | 1 | VUS |
| *CDH1* | NM_004360.3 | Chr16(GRCh37):g.68835670G>C | 3/16 | c.261G>C | p.(Arg87Ser) | / | Class3 | missense | 1 | VUS |
| *CDH1* | NM_004360.3 | Chr16(GRCh37):g.68835711A>G | 3/16 | c.302A>G | p.(Tyr101Cys) | / | Class3 | missense | 1 | VUS |
| *CDH1* | NM_004360.3 | Chr16(GRCh37):g.68835753C>T | 3/16 | c.344C>T | p.(Thr115Met) | / | Class3 | missense | 2 | VUS |
| *CDH1* | NM_004360.3 | Chr16(GRCh37):g.68835776C>G | 3/16 | c.367C>G | p.(His123Asp) | / | Class3 | missense | 1 | VUS |
| *CDH1* | NM_004360.3 | Chr16(GRCh37):g.68842376C>G | 4/16 | c.437C>G | p.(Ser146Cys) | / | Class3 | missense | 1 | VUS |
| *CDH1* | NM_004360.3 | Chr16(GRCh37):g.68842734C>T | 5/16 | c.670C>T | p.(Arg224Cys) | / | Class3 | missense | 4 | VUS |
| *CDH1* | NM_004360.3 | Chr16(GRCh37):g.68842735G>A | 5/16 | c.671G>A | p.(Arg224His) | / | Class3 | missense | 1 | VUS |
| *CDH1* | NM_004360.3 | Chr16(GRCh37):g.68844193G>A | 6/16 | c.781G>A | p.(Glu261Lys) | / | Class3 | missense | 1 | VUS |
| *CDH1* | NM_004360.3 | Chr16(GRCh37):g.68844196T>G | 6/16 | c.784T>G | p.(Phe262Val) | / | Class3 | missense | 1 | VUS |
| *CDH1* | NM_004360.3 | Chr16(GRCh37):g.68844220T>G | 6/16 | c.808T>G | p.(Ser270Ala) | CM014330 | Class3 | missense | 1 | VUS |
| *CDH1* | NM_004360.3 | Chr16(GRCh37):g.68845646G>A | 7/16 | c.892G>A | p.(Ala298Thr) | CM041745 | Class3 | missense | 5 | VUS |
| *CDH1* | NM_004360.3 | Chr16(GRCh37):g.68845661A>T | 7/16 | c.907A>T | p.(Thr303Ser) | / | Class3 | missense | 1 | VUS |
| *CDH1* | NM_004360.3 | Chr16(GRCh37):g.68845693C>G | 7/16 | c.939C>G | p.(Asp313Glu) | / | Class3 | missense | 1 | VUS |
| *CDH1* | NM_004360.3 | Chr16(GRCh37):g.68846080C>G | 8/16 | c.1051C>G | p.(Gln351Glu) | / | Class3 | missense | 1 | VUS |
| *CDH1* | NM_004360.3 | Chr16(GRCh37):g.68846132C>G | 8/16 | c.1103C>G | p.(Thr368Ser) | / | Class3 | missense | 1 | VUS |
| **gene** | **NCBI accession** | **genomic coordinates** | **exon/intron** | **HGVS (nucleotide)** | **HGVS (protein)** | **HGMD** | **classification** | **consequence** | **no. of patients** | **category** |
| *CDH1* | NM_004360.3 | Chr16(GRCh37):g.68846147C>T | 8/16 | c.1118C>T | p.(Pro373Leu) | CM072928 | Class3 | missense | 1 | VUS |
| *CDH1* | NM_004360.3 | Chr16(GRCh37):g.68847292A>G | 9/16 | c.1214A>G | p.(Asn405Ser) | / | Class3 | missense | 2 | VUS |
| *CDH1* | NM_004360.3 | Chr16(GRCh37):g.68847351G>A | 9/16 | c.1273G>A | p.(Val425Ile) | / | Class3 | missense | 2 | VUS |
| *CDH1* | NM_004360.3 | Chr16(GRCh37):g.68849514G>A | 10/16 | c.1417G>A | p.(Val473Ile) | / | Class3 | missense | 1 | VUS |
| *CDH1* | NM_004360.3 | Chr16(GRCh37):g.68849565G>A | 10/16 | c.1468G>A | p.(Glu490Lys) | / | Class3 | missense | 1 | VUS |
| *CDH1* | NM_004360.3 | Chr16(GRCh37):g.68849574G>C | 10/16 | c.1477G>C | p.(Val493Leu) | / | Class3 | missense | 1 | VUS |
| *CDH1* | NM_004360.3 | Chr16(GRCh37):g.68853185A>G | 11/16 | c.1568A>G | p.(Tyr523Cys) | / | Class3 | missense | 1 | VUS |
| *CDH1* | NM_004360.3 | Chr16(GRCh37):g.68855958A>G | 12/16 | c.1766A>G | p.(Asn589Ser) | / | Class3 | missense | 1 | VUS |
| *CDH1* | NM_004360.3 | Chr16(GRCh37):g.68857353A>G | 13/16 | c.1988A>G | p.(Tyr663Cys) | / | Class3 | missense | 3 | VUS |
| *CDH1* | NM_004360.3 | Chr16(GRCh37):g.68857398T>C | 13/16 | c.2033T>C | p.(Val678Ala) | / | Class3 | missense | 1 | VUS |
| *CDH1* | NM_004360.3 | Chr16(GRCh37):g.68862116C>T | 14/16 | c.2204C>T | p.(Ala735Val) | / | Class3 | missense | 1 | VUS |
| *CDH1* | NM_004360.3 | Chr16(GRCh37):g.68862157C>T | 14/16 | c.2245C>T | p.(Arg749Trp) | CM072929 | Class3 | missense | 1 | VUS |
| *CDH1* | NM_004360.3 | Chr16(GRCh37):g.68862158G>A | 14/16 | c.2246G>A | p.(Arg749Gln) | / | Class3 | missense | 1 | VUS |
| *CDH1* | NM_004360.3 | Chr16(GRCh37):g.68863590G>A | 15/16 | c.2329G>A | p.(Asp777Asn) | CM022776 | Class3 | missense | 1 | VUS |
| *CDH1* | NM_004360.3 | Chr16(GRCh37):g.68863597G>A | 15/16 | c.2336G>A | p.(Arg779Gln) | / | Class3 | missense | 1 | VUS |
| *CDH1* | NM_004360.3 | Chr16(GRCh37):g.68863604A>T | 15/16 | c.2343A>T | p.(Glu781Asp) | CM072930 | Class3 | missense | 2 | VUS |
| *CDH1* | NM_004360.3 | Chr16(GRCh37):g.68863617G>A | 15/16 | c.2356G>A | p.(Asp786Asn) | / | Class3 | missense | 1 | VUS |
| *CDH1* | NM_004360.3 | Chr16(GRCh37):g.68863629A>G | 15/16 | c.2368A>G | p.(Thr790Ala) | / | Class3 | missense | 1 | VUS |
| *CDH1* | NM_004360.3 | Chr16(GRCh37):g.68863648G>A | 15/16 | c.2387G>A | p.(Arg796Gln) | / | Class3 | missense | 2 | VUS |
| *CDH1* | NM_004360.3 | Chr16(GRCh37):g.68863674G>A | 15/16 | c.2413G>A | p.(Asp805Asn) | CM130989 | Class3 | missense | 2 | VUS |
| *CDH1* | NM_004360.3 | Chr16(GRCh37):g.68867247G>A | 16/16 | c.2494G>A | p.(Val832Met) | CM023731 | Class3 | missense | 1 | VUS |
| *CDH1* | NM_004360.3 | Chr16(GRCh37):g.68867265A>G | 16/16 | c.2512A>G | p.(Ser838Gly) | CM1612919 | Class3 | missense | 6 | VUS |
| *CDH1* | NM_004360.3 | Chr16(GRCh37):g.68867329A>G | 16/16 | c.2576A>G | p.(Tyr859Cys) | / | Class3 | missense | 1 | VUS |
| *CDH1* | NM_004360.3 | Chr16(GRCh37):g.68867388G>A | 16/16 | c.2635G>A | p.(Gly879Ser) | / | Class3 | missense | 3 | VUS |
| *CHEK2* | NM_007194.3 | Chr22(GRCh37):g.29130717T>C | intron 1 | c.-6-2A>G | p.(?) | / | Class3 | splice defect?* | 1 | VUS |
| *CHEK2* | NM_007194.3 | Chr22(GRCh37):g.29130713G>A | 2/15 | c.-4C>T | p.(?) | CR068233 | Class3 | splice defect?, 3^rd^ nt. | 2 | VUS |
| *CHEK2* | NM_007194.3 | Chr22(GRCh37):g.29130696G>A | 2/15 | c.14C>T | p.(Ser5Leu) | / | Class3 | missense | 1 | VUS |
| *CHEK2* | NM_007194.3 | Chr22(GRCh37):g.29130520C>T | 2/15 | c.190G>A | p.(Glu64Lys) | CM030414 | Class3 | missense | 3 | VUS |
| *CHEK2* | NM_007194.3 | Chr22(GRCh37):g.29130495T>C | 2/15 | c.215A>G | p.(Tyr72Cys) | / | Class3 | missense | 1 | VUS |
| *CHEK2* | NM_007194.3 | Chr22(GRCh37):g.29130450_29130464del | 2/15 | c.246_260del | p.(Asp82_Glu86del) | CD030580 | Class3 | inframe deletion | 1 | VUS |
| *CHEK2* | NM_007194.3 | Chr22(GRCh37):g.29130456G>A | 2/15 | c.254C>T | p.(Pro85Leu) | CM077521 | Class3 | missense | 1 | VUS |
| *CHEK2* | NM_007194.3 | Chr22(GRCh37):g.29130403A>G | 2/15 | c.307T>C | p.(Phe103Leu) | / | Class3 | missense | 1 | VUS |
| *CHEK2* | NM_007194.3 | Chr22(GRCh37):g.29121353A>G | 3/15 | c.322T>C | p.(Cys108Arg) | / | Class3 | missense | 1 | VUS |
| *CHEK2* | NM_007194.3 | Chr22(GRCh37):g.29121329C>T | 3/15 | c.346G>A | p.(Gly116Arg) | / | Class3 | missense | 1 | VUS |
| *CHEK2* | NM_007194.3 | Chr22(GRCh37):g.29121265C>T | 3/15 | c.410G>A | p.(Arg137Gln) | CM023899 | Class3 | missense | 2 | VUS |
| *CHEK2* | NM_007194.3 | Chr22(GRCh37):g.29121253T>G | 3/15 | c.422A>C | p.(Lys141Thr) | CM1612922 | Class3 | missense | 1 | VUS |
| *CHEK2* | NM_007194.3 | Chr22(GRCh37):g.29121241C>T | 3/15 | c.434G>A | p.(Arg145Gln) | CM092017 | Class3 | missense | 1 | VUS |
| *CHEK2* | NM_007194.3 | Chr22(GRCh37):g.29121228T>C | intron3 | c.444+3A>G | p.(?) | / | Class3 | splice defect? | 1 | VUS |
| *CHEK2* | NM_007194.3 | Chr22(GRCh37):g.29121212A>G | intron3 | c.444+19T>C | p.(?) | / | Class3 | splice defect? | 1 | VUS |
| *CHEK2* | NM_007194.3 | Chr22(GRCh37):g.29121087A>G | 4/15 | c.470T>C | p.(Ile157Thr) | CM993368 | Class3 | missense | 86 | VUS |
| *CHEK2* | NM_007194.3 | Chr22(GRCh37):g.29121060T>C | 4/15 | c.497A>G | p.(Asn166Ser) | / | Class3 | missense | 1 | VUS |
| *CHEK2* | NM_007194.3 | Chr22(GRCh37):g.29121019G>A | 4/15 | c.538C>T | p.(Arg180Cys) | CM030417 | Class3 | missense | 13 | VUS |
| *CHEK2* | NM_007194.3 | Chr22(GRCh37):g.29121018C>T | 4/15 | c.539G>A | p.(Arg180His) | CM030418 | Class3 | missense | 3 | VUS |
| *CHEK2* | NM_007194.3 | Chr22(GRCh37):g.29121016G>A | 4/15 | c.541C>T | p.(Arg181Cys) | CM030419 | Class3 | missense | 4 | VUS |
| *CHEK2* | NM_007194.3 | Chr22(GRCh37):g.29120979A>G | 4/15 | c.578T>C | p.(Leu193Pro) | / | Class3 | missense | 1 | VUS |
| *CHEK2* | NM_007194.3 | Chr22(GRCh37):g.29115398G>A | 5/15 | c.668C>T | p.(Ser223Leu) | / | Class3 | missense | 1 | VUS |
| *CHEK2* | NM_007194.3 | Chr22(GRCh37):g.29108001C>G | 6/15 | c.688G>C | p.(Ala230Pro) | / | Class3 | missense | 2 | VUS |
| *CHEK2* | NM_007194.3 | Chr22(GRCh37):g.29107994C>T | 6/15 | c.695G>A | p.(Gly232Glu) | / | Class3 | missense | 2 | VUS |
| *CHEK2* | NM_007194.3 | Chr22(GRCh37):g.29107974C>T | 6/15 | c.715G>A | p.(Glu239Lys) | CM030421 | Class3 | missense | 2 | VUS |
| *CHEK2* | NM_007194.3 | Chr22(GRCh37):g.29107938T>A | 6/15 | c.751A>T | p.(Ile251Phe) | CM030423 | Class3 | missense | 1 | VUS |
| *CHEK2* | NM_007194.3 | Chr22(GRCh37):g.29099495T>G | 8/15 | c.906A>C | p.(Glu302Asp) | / | Class3 | missense | 1 | VUS |
| *CHEK2* | NM_007194.3 | Chr22(GRCh37):g.29099489T>C | intron8 | c.908+4A>G | p.(?) | / | Class3 | splice defect? | 1 | VUS |
| *CHEK2* | NM_007194.3 | Chr22(GRCh37):g.29099486T>C | intron8 | c.908+7A>G | p.(?) | / | Class3 | splice defect? | 1 | VUS |
| *CHEK2* | NM_007194.3 | Chr22(GRCh37):g.29095882G>A | 9/15 | c.952C>T | p.(Arg318Cys) | / | Class3 | missense | 1 | VUS |
| **gene** | **NCBI accession** | **genomic coordinates** | **exon/intron** | **HGVS (nucleotide)** | **HGVS (protein)** | **HGMD** | **classification** | **consequence** | **no. of patients** | **category** |
| *CHEK2* | NM_007194.3 | Chr22(GRCh37):g.29095861T>C | 9/15 | c.973A>G | p.(Lys325Glu) | / | Class3 | missense | 2 | VUS |
| *CHEK2* | NM_007194.3 | Chr22(GRCh37):g.29095854T>C | 9/15 | c.980A>G | p.(Tyr327Cys) | CM030426 | Class3 | missense | 3 | VUS |
| *CHEK2* | NM_007194.3 | Chr22(GRCh37):g.29095852A>T | 9/15 | c.982T>A | p.(Phe328Ile) | / | Class3 | missense | 1 | VUS |
| *CHEK2* | NM_007194.3 | Chr22(GRCh37):g.29092951G>A | 10/15 | c.1033C>T | p.(His345Tyr) | / | Class3 | missense | 1 | VUS |
| *CHEK2* | NM_007194.3 | Chr22(GRCh37):g.29092948G>A | 10/15 | c.1036C>T | p.(Arg346Cys) | CM110730 | Class3 | missense | 2 | VUS |
| *CHEK2* | NM_007194.3 | Chr22(GRCh37):g.29092947C>T | 10/15 | c.1037G>A | p.(Arg346His) | / | Class3 | missense | 1 | VUS |
| *CHEK2* | NM_007194.3 | Chr22(GRCh37):g.29092945C>T | 10/15 | c.1039G>A | p.(Asp347Asn) | CM1617608 | Class3 | missense | 2 | VUS |
| *CHEK2* | NM_007194.3 | Chr22(GRCh37):g.29092917G>A | 10/15 | c.1067C>T | p.(Ser356Leu) | CM1511946 | Class3 | missense | 1 | VUS |
| *CHEK2* | NM_007194.3 | Chr22(GRCh37):g.29092903C>G | 10/15 | c.1081G>C | p.(Asp361His) | / | Class3 | missense | 1 | VUS |
| *CHEK2* | NM_007194.3 | Chr22(GRCh37):g.29092893A>G | 10/15 | c.1091T>C | p.(Ile364Thr) | CM088200 | Class3 | missense | 2 | VUS |
| *CHEK2* | NM_007194.3 | Chr22(GRCh37):g.29091827T>C | 11/15 | c.1130A>G | p.(Glu377Gly) | / | Class3 | missense | 1 | VUS |
| *CHEK2* | NM_007194.3 | Chr22(GRCh37):g.29091804A>G | 11/15 | c.1153T>C | p.(Cys385Arg) | / | Class3 | missense | 1 | VUS |
| *CHEK2* | NM_007194.3 | Chr22(GRCh37):g.29091782G>A | 11/15 | c.1175C>T | p.(Ala392Val) | / | Class3 | missense | 2 | VUS |
| *CHEK2* | NM_007194.3 | Chr22(GRCh37):g.29091722C>T | 11/15 | c.1235G>A | p.(Ser412Asn) | / | Class3 | missense | 1 | VUS |
| *CHEK2* | NM_007194.3 | Chr22(GRCh37):g.29091207G>A | 12/15 | c.1283C>T | p.(Ser428Phe) | CM051021 | Class3 | missense | 1 | VUS |
| *CHEK2* | NM_007194.3 | Chr22(GRCh37):g.29091178C>A | 12/15 | c.1312G>T | p.(Asp438Tyr) | CM077522 | Class3 | missense | 9 | VUS |
| *CHEK2* | NM_007194.3 | Chr22(GRCh37):g.29091154T>C | 12/15 | c.1336A>G | p.(Asn446Asp) | CM1511948 | Class3 | missense | 4 | VUS |
| *CHEK2* | NM_007194.3 | Chr22(GRCh37):g.29090099T>C | 13/15 | c.1382A>G | p.(Asp461Gly) | / | Class3 | missense | 1 | VUS |
| *CHEK2* | NM_007194.3 | Chr22(GRCh37):g.29090089C>A | 13/15 | c.1392G>T | p.(Lys464Asn) | / | Class3 | missense | 1 | VUS |
| *CHEK2* | NM_007194.3 | Chr22(GRCh37):g.29090080_29090082del | 13/15 | c.1399_1401del | p.(Leu467del) | / | Class3 | inframe deletion | 2 | VUS |
| *CHEK2* | NM_007194.3 | Chr22(GRCh37):g.29090073C>G | 13/15 | c.1408G>C | p.(Asp470His) | / | Class3 | missense | 1 | VUS |
| *CHEK2* | NM_007194.3 | Chr22(GRCh37):g.29090053_29090064del | 13/15 | c.1417_1428del | p.(Ala473_Thr476del) | / | Class3 | inframe deletion | 1 | VUS |
| *CHEK2* | NM_007194.3 | Chr22(GRCh37):g.29090061G>A | 13/15 | c.1420C>T | p.(Arg474Cys) | CM1614814 | Class3 | missense | 2 | VUS |
| *CHEK2* | NM_007194.3 | Chr22(GRCh37):g.29090060C>T | 13/15 | c.1421G>A | p.(Arg474His) | CM1511949 | Class3 | missense | 3 | VUS |
| *CHEK2* | NM_007194.3 | Chr22(GRCh37):g.29090015C>T | intron13 | c.1461+5G>A | p.(?) | / | Class3 | splice defect? | 1 | VUS |
| *CHEK2* | NM_007194.3 | Chr22(GRCh37):g.29085140G>A | 14/15 | c.1525C>T | p.(Pro509Ser) | / | Class3 | missense | 2 | VUS |
| *CHEK2* | NM_007194.3 | Chr22(GRCh37):g.29083961C>T | 15/15 | c.1556G>A | p.(Arg519Gln) | / | Class3 | missense | 1 | VUS |
| *CHEK2* | NM_007194.3 | Chr22(GRCh37):g.29083961C>A | 15/15 | c.1556G>T | p.(Arg519Leu) | / | Class3 | missense | 2 | VUS |
| *CHEK2* | NM_007194.3 | Chr22(GRCh37):g.29083956G>A | 15/15 | c.1561C>T | p.(Arg521Trp) | CM1614653 | Class3 | missense | 3 | VUS |
| *CHEK2* | NM_007194.3 | Chr22(GRCh37):g.29083926C>A | 15/15 | c.1591G>T | p.(Glu531*) | / | Class3 | nonsense, 39nt/13aa upstream of the stop codon | 1 | VUS |
| *CHEK2* | NM_007194.3 | Chr22(GRCh37):g.29083920T>C | 15/15 | c.1597A>G | p.(Thr533Ala) | / | Class3 | missense | 1 | VUS |
| *NBN* | NM_002485.4 | Chr8(GRCh37):g.90996750T>C | intron1 | c.37+3A>G | p.(?) | / | Class3 | splice defect? | 1 | VUS |
| *NBN* | NM_002485.4 | Chr8(GRCh37):g.90995048C>T | 2/16 | c.73G>A | p.(Val25Ile) | / | Class3 | missense | 3 | VUS |
| *NBN* | NM_002485.4 | Chr8(GRCh37):g.90995002G>A | 2/16 | c.119C>T | p.(Ser40Leu) | / | Class3 | missense | 1 | VUS |
| *NBN* | NM_002485.4 | Chr8(GRCh37):g.90993645G>A | 3/16 | c.278C>T | p.(Ser93Leu) | / | Class3 | missense | 2 | VUS |
| *NBN* | NM_002485.4 | Chr8(GRCh37):g.90993639T>C | 3/16 | c.284A>G | p.(Asp95Gly) | / | Class3 | missense | 2 | VUS |
| *NBN* | NM_002485.4 | Chr8(GRCh37):g.90993099C>T | 4/16 | c.343G>A | p.(Ala115Thr) | / | Class3 | missense | 1 | VUS |
| *NBN* | NM_002485.4 | Chr8(GRCh37):g.90993092G>A | 4/16 | c.350C>T | p.(Ser117Phe) | / | Class3 | missense | 1 | VUS |
| *NBN* | NM_002485.4 | Chr8(GRCh37):g.90990527G>A | 5/16 | c.505C>T | p.(Arg169Cys) | / | Class3 | missense | 1 | VUS |
| *NBN* | NM_002485.4 | Chr8(GRCh37):g.90990526C>T | 5/16 | c.506G>A | p.(Arg169His) | / | Class3 | missense | 2 | VUS |
| *NBN* | NM_002485.4 | Chr8(GRCh37):g.90990521T>C | 5/16 | c.511A>G | p.(lle171Val) | CM011800 | Class3 | missense | 34 | VUS |
| *NBN* | NM_002485.4 | Chr8(GRCh37):g.90990513C>G | 5/16 | c.519G>C | p.(Lys173Asn) | / | Class3 | missense | 1 | VUS |
| *NBN* | NM_002485.4 | Chr8(GRCh37):g.90983508G>A | 6/16 | c.595C>T | p.(Pro199Ser) | CM123120 | Class3 | missense | 1 | VUS |
| *NBN* | NM_002485.4 | Chr8(GRCh37):g.90983507G>C | 6/16 | c.596C>G | p.(Pro199Arg) | / | Class3 | missense | 5 | VUS |
| *NBN* | NM_002485.4 | Chr8(GRCh37):g.90983475C>A | 6/16 | c.628G>T | p.(Val210Phe) | / | Class3 | missense | 10 | VUS |
| *NBN* | NM_002485.4 | Chr8(GRCh37):g.90983470A>T | 6/16 | c.633T>A | p.(Asp211Glu) | CM1514927 | Class3 | missense | 1 | VUS |
| *NBN* | NM_002485.4 | Chr8(GRCh37):g.90983439A>G | 6/16 | c.664T>C | p.(Phe222Leu) | CM076331 | Class3 | missense | 1 | VUS |
| *NBN* | NM_002485.4 | Chr8(GRCh37):g.90983432C>T | 6/16 | c.671G>A | p.(Gly224Glu) | / | Class3 | missense | 1 | VUS |
| *NBN* | NM_002485.4 | Chr8(GRCh37):g.90982713C>T | 7/16 | c.775G>A | p.(Glu259Lys) | / | Class3 | missense | 1 | VUS |
| *NBN* | NM_002485.4 | Chr8(GRCh37):g.90982685G>A | 7/16 | c.803C>T | p.(Thr268Met) | CM1512711 | Class3 | missense | 3 | VUS |
| *NBN* | NM_002485.4 | Chr8(GRCh37):g.90982608T>A | 7/16 | c.880A>T | p.(Met294Leu) | / | Class3 | missense | 2 | VUS |
| *NBN* | NM_002485.4 | Chr8(GRCh37):g.90967646A>G | 10/16 | c.1262T>C | p.(Leu421Ser) | / | Class3 | missense | 6 | VUS |
| *NBN* | NM_002485.4 | Chr8(GRCh37):g.90967539T>C | 10/16 | c.1369A>G | p.(Asn457Asp) | / | Class3 | missense | 1 | VUS |
| **gene** | **NCBI accession** | **genomic coordinates** | **exon/intron** | **HGVS (nucleotide)** | **HGVS (protein)** | **HGMD** | **classification** | **consequence** | **no. of patients** | **category** |
| *NBN* | NM_002485.4 | Chr8(GRCh37):g.90965900G>T | 11/16 | c.1417C>A | p.(Gln473Lys) | / | Class3 | missense | 1 | VUS |
| *NBN* | NM_002485.4 | Chr8(GRCh37):g.90965695G>C | 11/16 | c.1622C>G | p.(Ala541Gly) | / | Class3 | missense | 1 | VUS |
| *NBN* | NM_002485.4 | Chr8(GRCh37):g.90965658C>T | 11/16 | c.1659G>A | p.(Met553Ile) | / | Class3 | missense | 1 | VUS |
| *NBN* | NM_002485.4 | Chr8(GRCh37):g.90965650A>T | 11/16 | c.1667T>A | p.(Val556Glu) | / | Class3 | missense | 1 | VUS |
| *NBN* | NM_002485.4 | Chr8(GRCh37):g.90965579C>T | 11/16 | c.1738G>A | p.(Val580Ile) | / | Class3 | missense | 1 | VUS |
| *NBN* | NM_002485.4 | Chr8(GRCh37):g.90958519T>C | 13/16 | c.1919A>G | p.(Asn640Ser) | / | Class3 | missense | 1 | VUS |
| *NBN* | NM_002485.4 | Chr8(GRCh37):g.90958513T>C | 13/16 | c.1925A>G | p.(Lys642Arg) | / | Class3 | missense | 1 | VUS |
| *NBN* | NM_002485.4 | Chr8(GRCh37):g.90958505C>A | 13/16 | c.1933G>T | p.(Asp645Tyr) | / | Class3 | missense | 1 | VUS |
| *NBN* | NM_002485.4 | Chr8(GRCh37):g.90958417C>T | 13/16 | c.2021G>A | p.(Gly674Asp) | / | Class3 | missense | 1 | VUS |
| *NBN* | NM_002485.4 | Chr8(GRCh37):g.90958411T>C | 13/16 | c.2027A>G | p.(Asn676Ser) | / | Class3 | missense | 1 | VUS |
| *NBN* | NM_002485.4 | Chr8(GRCh37):g.90958409C>T | 13/16 | c.2029G>A | p.(Asp677Asn) | / | Class3 | missense | 1 | VUS |
| *NBN* | NM_002485.4 | Chr8(GRCh37):g.90955557A>G | 14/16 | c.2108T>C | p.(Ile703Thr) | / | Class3 | missense | 1 | VUS |
| *NBN* | NM_002485.4 | Chr8(GRCh37):g.90955516T>A | 14/16 | c.2149A>T | p.(Thr717Ser) | / | Class3 | missense | 1 | VUS |
| *NBN* | NM_002485.4 | Chr8(GRCh37):g.90949287G>A | 15/16 | c.2201C>T | p.(Ala734Val) | / | Class3 | missense | 1 | VUS |
| *PALB2* | NM_024675.3 | Chr16(GRCh37):g.23652466G>A | 1/13 | c.13C>T | p.(Pro5Ser) | CM118266 | Class3 | missense | 1 | VUS |
| *PALB2* | NM_024675.3 | Chr16(GRCh37):g.23652442C>T | 1/13 | c.37G>A | p.(Glu13Lys) | CM1612930 | Class3 | missense | 1 | VUS |
| *PALB2* | NM_024675.3 | Chr16(GRCh37):g.23649414T>C | 2/13 | c.85A>G | p.(Ser29Gly) | CM1612931 | Class3 | missense | 1 | VUS |
| *PALB2* | NM_024675.3 | Chr16(GRCh37):g.23649399G>A | 2/13 | c.100C>T | p.(Arg34Cys) | / | Class3 | missense | 1 | VUS |
| *PALB2* | NM_024675.3 | Chr16(GRCh37):g.23649273G>T | 3/13 | c.109C>A | p.(Arg37Ser) | CM173433 | Class3 | missense | 1 | VUS |
| *PALB2* | NM_024675.3 | Chr16(GRCh37):g.23649234T>C | 3/13 | c.148A>G | p.(Lys50Glu) | / | Class3 | missense | 1 | VUS |
| *PALB2* | NM_024675.3 | Chr16(GRCh37):g.23649188G>A | 3/13 | c.194C>T | p.(Pro65Leu) | / | Class3 | missense | 1 | VUS |
| *PALB2* | NM_024675.3 | Chr16(GRCh37):g.23647641T>C | 4/13 | c.226A>G | p.(Ile76Val) | / | Class3 | missense | 1 | VUS |
| *PALB2* | NM_024675.3 | Chr16(GRCh37):g.23647638A>G | 4/13 | c.229T>C | p.(Cys77Arg) | / | Class3 | missense | 1 | VUS |
| *PALB2* | NM_024675.3 | Chr16(GRCh37):g.23647635C>T | 4/13 | c.232G>A | p.(Val78Ile) | CM1314575 | Class3 | missense | 2 | VUS |
| *PALB2* | NM_024675.3 | Chr16(GRCh37):g.23647518G>T | 4/13 | c.349C>A | p.(Pro117Thr) | / | Class3 | missense | 1 | VUS |
| *PALB2* | NM_024675.3 | Chr16(GRCh37):g.23647473C>T | 4/13 | c.394G>A | p.(Val132Ile) | / | Class3 | missense | 1 | VUS |
| *PALB2* | NM_024675.3 | Chr16(GRCh37):g.23647386C>G | 4/13 | c.481G>C | p.(Asp161His) | / | Class3 | missense | 2 | VUS |
| *PALB2* | NM_024675.3 | Chr16(GRCh37):g.23647310T>C | 4/13 | c.557A>G | p.(Asn186Ser) | / | Class3 | missense | 1 | VUS |
| *PALB2* | NM_024675.3 | Chr16(GRCh37):g.23647211T>C | 4/13 | c.656A>G | p.(Asp219Gly) | CM1314596 | Class3 | missense | 1 | VUS |
| *PALB2* | NM_024675.3 | Chr16(GRCh37):g.23647151C>A | 4/13 | c.716G>T | p.(Arg239Ile) | / | Class3 | missense | 1 | VUS |
| *PALB2* | NM_024675.3 | Chr16(GRCh37):g.23646968G>A | 4/13 | c.899C>T | p.(Thr300Ile) | CM108903 | Class3 | missense | 1 | VUS |
| *PALB2* | NM_024675.3 | Chr16(GRCh37):g.23646866T>C | 4/13 | c.1001A>G | p.(Tyr334Cys) | / | Class3 | missense | 2 | VUS |
| *PALB2* | NM_024675.3 | Chr16(GRCh37):g.23646794G>T | 4/13 | c.1073C>A | p.(Pro358His) | / | Class3 | missense | 1 | VUS |
| *PALB2* | NM_024675.3 | Chr16(GRCh37):g.23646770T>C | 4/13 | c.1097A>G | p.(Asn366Ser) | / | Class3 | missense | 1 | VUS |
| *PALB2* | NM_024675.3 | Chr16(GRCh37):g.23646739A>C | 4/13 | c.1128T>G | p.(Ser376Arg) | / | Class3 | missense | 2 | VUS |
| *PALB2* | NM_024675.3 | Chr16(GRCh37):g.23646617G>T | 4/13 | c.1250C>A | p.(Ser417Tyr) | / | Class3 | missense | 6 | VUS |
| *PALB2* | NM_024675.3 | Chr16(GRCh37):g.23646575C>T | 4/13 | c.1292G>A | p.(Ser431Asn) | / | Class3 | missense | 1 | VUS |
| *PALB2* | NM_024675.3 | Chr16(GRCh37):g.23646419G>A | 4/13 | c.1448C>T | p.(Ser483Leu) | / | Class3 | missense | 1 | VUS |
| *PALB2* | NM_024675.3 | Chr16(GRCh37):g.23646341C>T | 4/13 | c.1526G>A | p.(Gly509Asp) | / | Class3 | missense | 1 | VUS |
| *PALB2* | NM_024675.3 | Chr16(GRCh37):g.23646323T>C | 4/13 | c.1544A>G | p.(Lys515Arg) | CM1612928 | Class3 | missense | 3 | VUS |
| *PALB2* | NM_024675.3 | Chr16(GRCh37):g.23646266G>C | 4/13 | c.1601C>G | p.(Ser534Cys) | / | Class3 | missense | 2 | VUS |
| *PALB2* | NM_024675.3 | Chr16(GRCh37):g.23641771T>G | 5/13 | c.1704A>C | p.(Gln568His) | / | Class3 | missense | 1 | VUS |
| *PALB2* | NM_024675.3 | Chr16(GRCh37):g.23641727A>C | 5/13 | c.1748T>G | p.(Leu583Trp) | / | Class3 | missense | 1 | VUS |
| *PALB2* | NM_024675.3 | Chr16(GRCh37):g.23641536G>C | 5/13 | c.1939C>G | p.(His647Asp) | / | Class3 | missense | 1 | VUS |
| *PALB2* | NM_024675.3 | Chr16(GRCh37):g.23641427T>A | 5/13 | c.2048A>T | p.(His683Leu) | / | Class3 | missense | 1 | VUS |
| *PALB2* | NM_024675.3 | Chr16(GRCh37):g.23641370A>T | 5/13 | c.2105T>A | p.(Ile702Lys) | / | Class3 | missense | 1 | VUS |
| *PALB2* | NM_024675.3 | Chr16(GRCh37):g.23641369T>C | 5/13 | c.2106A>G | p.(Ile702Met) | / | Class3 | missense | 1 | VUS |
| *PALB2* | NM_024675.3 | Chr16(GRCh37):g.23641346G>A | 5/13 | c.2129C>T | p.(Thr710Met) | / | Class3 | missense | 1 | VUS |
| *PALB2* | NM_024675.3 | Chr16(GRCh37):g.23641340G>A | 5/13 | c.2135C>T | p.(Ala712Val) | / | Class3 | missense | 2 | VUS |
| *PALB2* | NM_024675.3 | Chr16(GRCh37):g.23641150T>G | 5/13 | c.2325A>C | p.(Gln775His) | / | Class3 | missense | 1 | VUS |
| *PALB2* | NM_024675.3 | Chr16(GRCh37):g.23637663C>T | 7/13 | c.2642G>A | p.(Gly881Asp) | / | Class3 | missense | 1 | VUS |
| *PALB2* | NM_024675.3 | Chr16(GRCh37):g.23635414A>G | 8/13 | c.2750T>C | p.(Val917Ala) | / | Class3 | missense | 2 | VUS |
| *PALB2* | NM_024675.3 | Chr16(GRCh37):g.23635412G>A | 8/13 | c.2752C>T | p.(Pro918Ser) | CM118268 | Class3 | missense | 1 | VUS |
| *PALB2* | NM_024675.3 | Chr16(GRCh37):g.23635391C>G | 8/13 | c.2773G>C | p.(Val925Leu) | / | Class3 | missense | 1 | VUS |
| **gene** | **NCBI accession** | **genomic coordinates** | **exon/intron** | **HGVS (nucleotide)** | **HGVS (protein)** | **HGMD** | **classification** | **consequence** | **no. of patients** | **category** |
| *PALB2* | NM_024675.3 | Chr16(GRCh37):g.23635372A>C | 8/13 | c.2792T>G | p.(Leu931Arg) | CM1516510 | Class3 | missense | 1 | VUS |
| *PALB2* | NM_024675.3 | Chr16(GRCh37):g.23635343T>C | 8/13 | c.2821A>G | p.(Ile941Val) | / | Class3 | missense | 1 | VUS |
| *PALB2* | NM_024675.3 | Chr16(GRCh37):g.23634404A>G | 9/13 | c.2882T>C | p.(Leu961Pro) | / | Class3 | missense | 1 | VUS |
| *PALB2* | NM_024675.3 | Chr16(GRCh37):g.23634383G>C | 9/13 | c.2903C>G | p.(Ala968Gly) | / | Class3 | missense | 1 | VUS |
| *PALB2* | NM_024675.3 | Chr16(GRCh37):g.23632742C>G | 10/13 | c.3054G>C | p.(Glu1018Asp) | CM118269 | Class3 | missense | 1 | VUS |
| *PALB2* | NM_024675.3 | Chr16(GRCh37):g.23632723C>T | 10/13 | c.3073G>A | p.(Ala1025Thr) | / | Class3 | missense | 2 | VUS |
| *PALB2* | NM_024675.3 | Chr16(GRCh37):g.23625378G>T | 11/13 | c.3148C>A | p.(His1050Asn) | / | Class3 | missense | 1 | VUS |
| *PALB2* | NM_024675.3 | Chr16(GRCh37):g.23619284G>A | 12/13 | c.3251C>T | p.(Ser1084Leu) | / | Class3 | missense | 2 | VUS |
| *PALB2* | NM_024675.3 | Chr16(GRCh37):g.23619228C>T | 12/13 | c.3307G>A | p.(Val1103Met) | CM118272 | Class3 | missense | 1 | VUS |
| *PALB2* | NM_024675.3 | Chr16(GRCh37):g.23619204G>C | 12/13 | c.3331C>G | p.(Pro1111Ala) | / | Class3 | missense | 1 | VUS |
| *PALB2* | NM_024675.3 | Chr16(GRCh37):g.23619193C>G | 12/13 | c.3342G>C | p.(Gln1114His) | / | Class3 | missense | 1 | VUS |
| *PALB2* | NM_024675.3 | Chr16(GRCh37):g.23614913A>T | 13/13 | c.3428T>A | p.(Leu1143His) | / | Class3 | missense | 1 | VUS |
| *PALB2* | NM_024675.3 | Chr16(GRCh37):g.23614908C>A | 13/13 | c.3433G>T | p.(Gly1145Cys) | / | Class3 | missense | 1 | VUS |
| *PALB2* | NM_024675.3 | Chr16(GRCh37):g.23614884G>C | 13/13 | c.3457C>G | p.(Pro1153Ala) | / | Class3 | missense | 1 | VUS |
| *PALB2* | NM_024675.3 | Chr16(GRCh37):g.23614847G>A | 13/13 | c.3494C>T | p.(Ser1165Leu) | / | Class3 | missense | 1 | VUS |
| *PALB2* | NM_024675.3 | Chr16(GRCh37):g.23614833G>A | 13/13 | c.3508C>T | p.(His1170Tyr) | / | Class3 | missense | 5 | VUS |
| *RAD51C* | NM_058216.2 | Chr17(GRCh37):g.56770011G>A | 1/9 | c.7G>A | p.(Gly3Arg) | / | Class3 | missense | 4 | VUS |
| *RAD51C* | NM_058216.2 | Chr17(GRCh37):g.56770033T>G | 1/9 | c.29T>G | p.(Met10Arg) | CM119967 | Class3 | missense | 1 | VUS |
| *RAD51C* | NM_058216.2 | Chr17(GRCh37):g.56770066C>T | 1/9 | c.62C>T | p.(Pro21Leu) | / | Class3 | missense | 1 | VUS |
| *RAD51C* | NM_058216.2 | Chr17(GRCh37):g.56770069C>T | 1/9 | c.65C>T | p.(Ala22Val) | / | Class3 | missense | 1 | VUS |
| *RAD51C* | NM_058216.2 | Chr17(GRCh37):g.56772304C>G | 2/9 | c.158C>G | p.(Ser53Cys) | / | Class3 | missense | 1 | VUS |
| *RAD51C* | NM_058216.2 | Chr17(GRCh37):g.56772339A>G | 2/9 | c.193A>G | p.(Arg65Gly) | / | Class3 | missense | 1 | VUS |
| *RAD51C* | NM_058216.2 | Chr17(GRCh37):g.56772382C>T | 2/9 | c.236C>T | p.(Ser79Phe) | / | Class3 | missense | 1 | VUS |
| *RAD51C* | NM_058216.2 | Chr17(GRCh37):g.56772454T>A | 2/9 | c.308T>A | p.(Phe103Tyr) | / | Class3 | missense | 1 | VUS |
| *RAD51C* | NM_058216.2 | Chr17(GRCh37):g.56772534G>A | 2/9 | c.388G>A | p.(Gly130Arg) | / | Class3 | missense | 1 | VUS |
| *RAD51C* | NM_058216.2 | Chr17(GRCh37):g.56772540A>C | 2/9 | c.394A>C | p.(Thr132Pro) | CM159272 | Class3 | missense | 1 | VUS |
| *RAD51C* | NM_058216.2 | Chr17(GRCh37):g.56772543C>A | 2/9 | c.397C>A | p.(Gln133Lys) | CM159273 | Class3 | missense | 1 | VUS |
| *RAD51C* | NM_058216.2 | Chr17(GRCh37):g.56774063G>C | 3/9 | c.414G>C | p.(Leu138Phe) | CM102527 | Class3 | missense | 1 | VUS |
| *RAD51C* | NM_058216.2 | Chr17(GRCh37):g.56774080T>C | 3/9 | c.431T>C | p.(Ile144Thr) | CM123222 | Class3 | missense | 2 | VUS |
| *RAD51C* | NM_058216.2 | Chr17(GRCh37):g.56774155T>C | 3/9 | c.506T>C | p.(Val169Ala) | / | Class3 | missense | 3 | VUS |
| *RAD51C* | NM_058216.2 | Chr17(GRCh37):g.56774169A>G | 3/9 | c.520A>G | p.(Thr174Ala) | / | Class3 | missense | 1 | VUS |
| *RAD51C* | NM_058216.2 | Chr17(GRCh37):g.56780598C>A | 4/9 | c.613C>A | p.(Leu205Ile) | CM1611045 | Class3 | missense | 1 | VUS |
| *RAD51C* | NM_058216.2 | Chr17(GRCh37):g.56780606T>A | 4/9 | c.621T>A | p.(His207Gln) | / | Class3 | missense | 2 | VUS |
| *RAD51C* | NM_058216.2 | Chr17(GRCh37):g.56780626G>A | 4/9 | c.641G>A | p.(Arg214His) | / | Class3 | missense | 1 | VUS |
| *RAD51C* | NM_058216.2 | Chr17(GRCh37):g.56780662_56780664del | 4/9 | c.677_679del | p.(Leu226del) | / | Class3 | inframe deletion | 1 | VUS |
| *RAD51C* | NM_058216.2 | Chr17(GRCh37):g.56787262C>T | 5/9 | c.748C>T | p.(His250Tyr) | / | Class3 | missense | 1 | VUS |
| *RAD51C* | NM_058216.2 | Chr17(GRCh37):g.56787286C>T | 5/9 | c.772C>T | p.(Arg258Cys) | / | Class3 | missense | 1 | VUS |
| *RAD51C* | NM_058216.2 | Chr17(GRCh37):g.56787298T>G | 5/9 | c.784T>G | p.(Leu262Val) | CM120337 | Class3 | missense | 2 | VUS |
| *RAD51C* | NM_058216.2 | Chr17(GRCh37):g.56787304G>A | 5/9 | c.790G>A | p.(Gly264Ser) | CM123214 | Class3 | missense | 56 | VUS |
| *RAD51C* | NM_058216.2 | Chr17(GRCh37):g.56801412G>A | 7/9 | c.916G>A | p.(Gly306Arg) | / | Class3 | missense | 1 | VUS |
| *RAD51D* | NM_002878.3 | Chr17(GRCh37):g.33446607C>G | 1/10 | c.26G>C | p.(Cys9Ser) | CM128416 | Class3 | missense | 1 | VUS |
| *RAD51D* | NM_002878.3 | Chr17(GRCh37):g.33446553G>A | 1/10 | c.80C>T | p.(Thr27Ile) | / | Class3 | missense | 1 | VUS |
| *RAD51D* | NM_002878.3 | Chr17(GRCh37):g.33445620G>A | 3/10 | c.163C>T | p.(Arg55Trp) | CM1612942 | Class3 | missense | 1 | VUS |
| *RAD51D* | NM_002878.3 | Chr17(GRCh37):g.33445616A>C | 3/10 | c.167T>G | p.(Val56Gly) | / | Class3 | missense | 1 | VUS |
| *RAD51D* | NM_002878.3 | Chr17(GRCh37):g.33445587C>T | 3/10 | c.196G>A | p.(Val66Met) | / | Class3 | missense | 1 | VUS |
| *RAD51D* | NM_002878.3 | Chr17(GRCh37):g.33445581C>T | 3/10 | c.202G>A | p.(Gly68Ser) | CM159278 | Class3 | missense | 1 | VUS |
| *RAD51D* | NM_002878.3 | Chr17(GRCh37):g.33434093C>T | 5/10 | c.394G>A | p.(Val132Ile) | CM1612944 | Class3 | missense | 1 | VUS |
| *RAD51D* | NM_002878.3 | Chr17(GRCh37):g.33433406T>C | 6/10 | c.575A>G | p.(Gln192Arg) | / | Class3 | missense | 1 | VUS |
| *RAD51D* | NM_002878.3 | Chr17(GRCh37):g.33430556C>T | 7/10 | c.584G>A | p.(Gly195Asp) | / | Class3 | missense | 1 | VUS |
| *RAD51D* | NM_002878.3 | Chr17(GRCh37):g.33430511G>T | 7/10 | c.629C>A | p.(Ala210Glu) | / | Class3 | missense | 1 | VUS |
| *RAD51D* | NM_002878.3 | Chr17(GRCh37):g.33430296G>A | 8/10 | c.715C>T | p.(Arg239Trp) | CM128414 | Class3 | missense | 3 | VUS |
| *RAD51D* | NM_002878.3 | Chr17(GRCh37):g.33428365C>T | 9/10 | c.758G>A | p.(Arg253Gln) | / | Class3 | missense | 1 | VUS |
| *RAD51D* | NM_002878.3 | Chr17(GRCh37):g.33428338G>A | 9/10 | c.785C>T | p.(Pro262Leu) | CM159283 | Class3 | missense | 1 | VUS |
| *RAD51D* | NM_002878.3 | Chr17(GRCh37):g.33428224C>T | 9/10 | c.899G>A | p.(Arg300Gln) | / | Class3 | missense | 2 | VUS |
| **gene** | **NCBI accession** | **genomic coordinates** | **exon/intron** | **HGVS (nucleotide)** | **HGVS (protein)** | **HGMD** | **classification** | **consequence** | **no. of patients** | **category** |
| *RAD51D* | NM_002878.3 | Chr17(GRCh37):g.33428027A>T | 10/10 | c.932T>A | p.(Ile311Asn) | CM128415 | Class3 | missense | 3 | VUS |
| *RAD51D* | NM_002878.3 | Chr17(GRCh37):g.33428015C>T | 10/10 | c.944G>A | p.(Gly315Glu) | / | Class3 | missense | 2 | VUS |
| *RAD51D* | NM_002878.3 | Chr17(GRCh37):g.33427988T>A | 10/10 | c.971A>T | p.(Gln324Leu) | / | Class3 | missense | 1 | VUS |
| *TP53* | NM_000546.5 | Chr17(GRCh37):g.7579887C>T | 2/11 | c.26G>A | p.(Ser9Asn) | / | Class3 | missense | 1 | VUS |
| *TP53* | NM_000546.5 | Chr17(GRCh37):g.7579884A>C | 2/11 | c.29T>G | p.(Val10Gly) | / | Class3 | missense | 1 | VUS |
| *TP53* | NM_000546.5 | Chr17(GRCh37):g.7579717G>A | 3/11 | c.79C>T | p.(Pro27Ser) | / | Class3 | missense | 1 | VUS |
| *TP53* | NM_000546.5 | Chr17(GRCh37):g.7579470C>T | 4/11 | c.217G>A | p.(Val73Met) | / | Class3 | missense | 1 | VUS |
| *TP53* | NM_000546.5 | Chr17(GRCh37):g.7579399_7579401del | 4/11 | c.286_288del | p.(Ser96del) | CD1511154 | Class3 | inframe deletion | 1 | VUS |
| *TP53* | NM_000546.5 | Chr17(GRCh37):g.7579358C>T | 4/11 | c.329G>A | p.(Arg110His) | CM134053 | Class3 | missense | 1 | VUS |
| *TP53* | NM_000546.5 | Chr17(GRCh37):g.7579334G>A | 4/11 | c.353C>T | p.(Thr118Ile) | / | Class3 | missense | 1 | VUS |
| *TP53* | NM_000546.5 | Chr17(GRCh37):g.7578556dup | intron4 | c.376-2dup | p.(?) | / | Class3 | splice defect? | 2 | VUS |
| *TP53* | NM_000546.5 | Chr17(GRCh37):g.7578530A>G | 5/11 | c.400T>C | p.(Phe134Leu) | / | Class3 | missense | 1 | VUS |
| *TP53* | NM_000546.5 | Chr17(GRCh37):g.7578460A>C | 5/11 | c.470T>G | p.(Val157Gly) | / | Class3 | missense | 1 | VUS |
| *TP53* | NM_000546.5 | Chr17(GRCh37):g.7578458G>C | 5/11 | c.472C>G | p.(Arg158Gly) | CM004341 | Class3 | missense | 1 | VUS |
| *TP53* | NM_000546.5 | Chr17(GRCh37):g.7578412A>C | 5/11 | c.518T>G | p.(Val173Gly) | / | Class3 | missense | 1 | VUS |
| *TP53* | NM_000546.5 | Chr17(GRCh37):g.7578407G>A | 5/11 | c.523C>T | p.(Arg175Cys) | CM118880 | Class3 | missense | 1 | VUS |
| *TP53* | NM_000546.5 | Chr17(GRCh37):g.7578204A>C | 6/11 | c.645T>G | p.(Ser215Arg) | / | Class3 | missense | 1 | VUS |
| *TP53* | NM_000546.5 | Chr17(GRCh37):g.7577596A>C | 7/11 | c.685T>G | p.(Cys229Gly) | / | Class3 | missense | 1 | VUS |
| *TP53* | NM_000546.5 | Chr17(GRCh37):g.7577577T>C | 7/11 | c.704A>G | p.(Asn235Ser) | CM951230 | Class3 | missense | 5 | VUS |
| *TP53* | NM_000546.5 | Chr17(GRCh37):g.7577504_7577518del | 7/11 | c.763_777del | p.(Ile255_Asp259del) | / | Class3 | inframe deletion | 1 | VUS |
| *TP53* | NM_000546.5 | Chr17(GRCh37):g.7577138C>G | 8/11 | c.800G>C | p.(Arg267Pro) | / | Class3 | missense | 1 | VUS |
| *TP53* | NM_000546.5 | Chr17(GRCh37):g.7577091G>A | 8/11 | c.847C>T | p.(Arg283Cys) | CM041458 | Class3 | missense | 1 | VUS |
| *TP53* | NM_000546.5 | Chr17(GRCh37):g.7577070G>A | 8/11 | c.868C>T | p.(Arg290Cys) | / | Class3 | missense | 1 | VUS |
| *TP53* | NM_000546.5 | Chr17(GRCh37):g.7577069C>T | 8/11 | c.869G>A | p.(Arg290His) | CM993905 | Class3 | missense | 3 | VUS |
| *TP53* | NM_000546.5 | Chr17(GRCh37):g.7577063T>C | 8/11 | c.875A>G | p.(Lys292Arg) | / | Class3 | missense | 1 | VUS |
| *TP53* | NM_000546.5 | Chr17(GRCh37):g.7577046C>T | 8/11 | c.892G>A | p.(Glu298Lys) | / | Class3 | missense | 1 | VUS |
| *TP53* | NM_000546.5 | Chr17(GRCh37):g.7576897G>T | 9/11 | c.949C>A | p.(Gln317Lys) | / | Class3 | missense | 1 | VUS |
| *TP53* | NM_000546.5 | Chr17(GRCh37):g.7576872C>T | 9/11 | c.974G>A | p.(Gly325Glu) | / | Class3 | missense | 1 | VUS |
| *TP53* | NM_000546.5 | Chr17(GRCh37):g.7573988C>T | 10/11 | c.1039G>A | p.(Ala347Thr) | / | Class3 | missense | 1 | VUS |
| *TP53* | NM_000546.5 | Chr17(GRCh37):g.7573954T>A | 10/11 | c.1073A>T | p.(Glu358Val) | / | Class3 | missense | 1 | VUS |
| *TP53* | NM_000546.5 | Chr17(GRCh37):g.7573948C>A | 10/11 | c.1079G>T | p.(Gly360Val) | / | Class3 | missense | 2 | VUS |
| *TP53* | NM_000546.5 | Chr17(GRCh37):g.7572958A>G | 11/11 | c.1151T>C | p.(Met384Thr) | / | Class3 | missense | 1 | VUS |
| *TP53* | NM_000546.5 | Chr17(GRCh37):g.7572938C>T | 11/11 | c.1171G>A | p.(Asp391Asn) | / | Class3 | missense | 1 | VUS |

**Table S2:** Class 3, 4 and 5 variants identified in the study sample of 5,589 BC index patients. For each variant, the genomic position, consequences on nucleotide and protein level according to the HGVS nomenclature and the HGMD accession number are given. Abbreviations: PTV = protein truncating variant; DV = deleterious variant; VUS = variant of uncertain significance. * For the *CHEK2* variant c.-6-2A>G consequence on splicing/protein level is unknown due to alternative 3’ splice sites adjacent to the canonical splice site in intron 1. We therefore classified this variant as a class 3 variant (VUS).

| **gene** | **cumulative carrier frequency ExAC (%)** | **cumulative carrier frequency BC cases (%)** | **OR (95%CI; P)** |
| --- | --- | --- | --- |
| *ATM* | *1.66* | *1.81* | *1.09 (0.88-1.36; 0.4265)* |
| *CDH1* | 0.42 | 0.41 | 0.98 (0.63-1.54; 0.9901) |
| *CHEK2* | **0.71** | **1.43** | 2.04 (1.57-2.65; <0.0001) |
| *NBN* | 0.17 | 0.13 | 0.74 (0.33-1.64; 0.5838) |
| *PALB2* | 0.25 | 0.34 | 1.36 (0.82-2.26; 0.2522) |
| *RAD51C* | 0.23 | 0.29 | 1.24 (0.71-2.14; 0.4536) |
| *RAD51D* | 0.09 | 0.18 | 1.95 (0.93-4.06; 0.0745) |
| *TP53* | **0.18** | **0.41** | 2.29 (1.39-3.76; 0.0024) |

**Table S3:** Prevalence of rare missense variants predicted to be damaging**.** Cumulative carrier frequency of potentially damaging, rare missense variants identified in the selected core genes. Missense variants were defined as potentially damaging when predicted to be deleterious by the *in silico* tools SIFT and MutationTaster (Alamut version 2.10 as of November 30^th^ 2017). Only rare variants (MAF<0.1% in ExAC, NFE excluding TCGA) were included in our analysis. A total of 1,008 individuals carrying potentially damaging missense variants in the 8 core genes were listed in the ExAC database (Exome Aggregation Consortium, non-Finnish Europeans; excluding The Cancer Genome Atlas data; as of June 2016); 245 individuals were listed in the FLOSSIES database (American-European ancestry) and 77 individuals were identified in geographically-matched female controls (GMCs); 279 individuals carrying potentially damaging missense variants were found in 5,589 BC index patients.
